# Cutaneous Diseases.—(Eclectic Article)

**Published:** 1835-10-01

**Authors:** 


### CUTANEOUS DISEASES.

1. A THEORETICAL AND PRACTICAL TREATISE ON DISEASES OF THE SKIN. By *P. Rayer*, M.D. Second Edit. 8vo, pp. 1238. London, 1835.

2. ATLAS OF 26 COLOURED ENGRAVINGS: Quarto.

3. A PRACTICAL COMPENDIUM OF THE DISEASES OF THE SKIN. By *Jonathan Green*, M.D. Octavo, pp. 371. London, 1835.

4. CYCLOPÆDIA OF PRACTICAL MEDICINE.—Various Articles.

In the Number of this Review for October, of last year, we submitted to our readers' attention a moderately extensive analytic notice of Rayer's Treatise on Cutaneous Diseases, availing ourselves of that opportunity to state freely the leading merits and demerits of the classification which he has proposed, and of the descriptions which he has given. We also commented on the manner in which the English translator, Mr. Dickenson, had performed his task, and pointed out to him the changes which ought to be made, in the event of his work arriving at a second edition. It may be remembered, that Mr. D. had considerably abridged the contents of the original, by condensing the narrative in several places, and by omitting the detailed reports of the numerous cases with which M. Rayer has illustrated his descriptions. The present translation, executed by Dr. Willis, contains the whole matter of the French edition. It is, therefore, a much larger work; and its value is greatly enhanced by the accompanying volume of coloured plates, which represent, with a most faithful accuracy, the appearance of every cutaneous disease. We are pleased that the work of Rayer has been, at length, presented to the English reader in its full amplitude and extent, for it is only in this way that the high value of this author's labours can be duly appreciated. The comprehensiveness of its design, the minute accuracy of its details, the admirable erudition and labour of research with which every topic has been treated, and, withal, the soundly-practical precepts which are every where inculcated, justly entitle it to the proud eminence of taking a place, in the ranks of medical literature, by the side of Good's Study of Medicine and of Cooper's Dictionary of Surgery.

The Practical Compendium of Dr. Green forms a popular and very useful introductory work to the larger one of Rayer. In the descriptions of most of the diseases, he has drawn largely, but not servilely, from the ample stores of his French compeer; illustrating, however, each subject, by frequent reference to the results of his own observation and experience, and dwelling more particularly on the striking efficacy of the various forms of baths, in the treatment of some of the most obstinate diseases which affect the skin. There can be no doubt that the remedial and curative effects of baths, simple and medicated, have been hitherto far too little appreciated in this country. The physicians on the Continent have always recognized their value more highly than we have done. Whether the neglect with which they have been treated by us, has arisen from the too general doctrine of referring almost all cutaneous diseases to derangements of the alimentary passages, or from the circumstance, that so many of the British quacks have, of late years, resorted to medicated baths as specifics (and, by the way, very profitable ones) for all and every ill that flesh is heir to, we need not tarry to enquire; but we are very certain that, on the one hand, the "stomach and bowel pathology" of our physicians has been carried to an extravagant and most pernicious extent, and that, on the other hand, not a few cases, which have long and most unhandsomely resisted all the therapeutic attacks of the *regulars*, have very readily yielded to a course of baths, under the *enlightened* superintendence of Mr. ——, in the City, and of Mr. ——, in the more fashionable neighbourhood of the West End. Indeed, the whole *manege* of these establishments is well designed for the *disinterested* views of the medicated-bath doctors. The patients usually go in the early part of the day, an hour or two after breakfast-time. The baths are fitted up in the most elegant and comfortable manner, and generally are rendered more efficacious (at least the patients must believe this) by their being impregnated with aromatic and other agreeable herbs. After being well steamed for a sufficient time, the body is briskly rubbed until a pleasant glow is induced. The clothes being put on, a dish of excellent coffee, served on a silver waiter, is brought to comfort the inner man! Now surely all this is infinitely better worth a fee than to be doctored with pills, draughts, and potions!!

Of a truth, these men of the world are wiser in their generation than the sons of legitimate science; and they have their reward, not only in a rich harvest of pounds, shillings, and pence, which annually replenish their garners, but also, it cannot be denied, in their occasional cures of some maladies which have defied the attacks of all the recognized contents of our pharmacopœias.

We are anxious to draw the attention of our brethren to this subject, and advise them to lay aside all feelings of mortified pride, if these really do exist, and to cease to undervalue a remedy merely because it is patronized by the irregular practitioner. The practical observations of Dr. Green will powerfully contribute to correct an error which has too long existed; and, indeed, no portion of the Compendium is so useful, as the illustrations which he has given of the admirable efficacy of well-regulated baths, in the treatment of skin diseases. The following extracts may be read with advantage, as preliminary to the treatment of individual diseases.

"The simple cold or tepid bath frequently gives great relief in many diseases of the skin, greatly allaying the itching, and state of nervous irritation that attend them, and thus conducing to the ultimate cure." "Baths of the natural mineral waters have been long known to prove very serviceable in many diseases of the skin. These are susceptible of being closely imitated by art; we can, indeed, in this way produce more powerful and more speedy effects than result from bathing in the natural mineral springs. The artificial sulphureous water bath, with a quantity of gelatine or fine glue dissolved in it, is one of the best baths known in many inveterate diseases of the skin.

But every form of water bathing that has been tried falls immeasurably short of the hot air and vapour bath, in its immediate and powerful curative influence on the great majority of the diseases of the skin. The hot air and vapour bath may very properly be spoken of together, inasmuch as their effects on the system are very nearly similar. I am in the habit of administering the hot air bath to patients at first, at the temperature of about 98° of Fahrenheit, and of raising it gradually in the course of from fifteen to twenty minutes to 110°, and, if the full effect of the bath is not obtained, to 120°, or even 130° of the same scale. The patient, seated in the apparatus and exposed to this degree of heat, is only sensible at first of a slightly increased but pleasant warmth. Within a few minutes the expression becomes cheerful and animated, the eyes sparkle, the countenance looks florid and then flushed, the pulse rises in frequency, and gains much in fulness, but is soft; the whole body, the face (which of course is not inclosed) as well as the other parts, next become bathed in perspiration, so that the sweat is seen standing in beads upon the forehead and trickling down the cheeks. The patient is now no longer sensible of any increase of temperature, although he is perhaps exposed to a heat of 150 degrees of Fahrenheit."

"After the perspiration has appeared about five or six minutes on the forehead, the full effect of the bath has been obtained, and the patient should immediately quit the apparatus." 20.

The therapeutic agency of the simple hot air and vapour baths (although these alone are often sufficient for the cure of many obstinate cutaneous diseases) is greatly promoted in particular cases, by their being impregnated with the fumes of certain substances, more especially of sulphur.

"This is now administered by a process of sublimation; the mineral is not set fire to and burned as used formerly to be done, and as is still practised in imperfectly constructed fumigating apparatuses, by which the patient is enveloped in an atmosphere of sulphurous acid gas, and runs considerable risk of being stifled each time he adventures himself within them. In my establishment, although twenty sulphur baths are frequently administered every day for weeks together, no smell of burning brimstone is ever perceptible. The mineral is raised unchanged, in a state of impalpable vapour, and is thus applied in a form which we may presume to be the most favourable possible, either for exerting its influence on the system at large, through the medium of the absorbent vessels, or upon the skin immediately, with which it is brought into such intimate contact. Applied in this way sulphur has certainly a greater curative influence on many of the inveterate and chronic diseases of the skin, than all other means besides when added together. Besides the powerful excitement of the skin accomplished by the application of a high temperature to its surface, the sulphur fumes have the property of causing a remarkable desquamation or peeling of the cuticle, to which probably some portion of their efficacy may justly be attributed." 21.

In our analytic review of the particular diseases, we shall meet with numerous illustrations of the truth of these remarks.

Dr. Green, although himself a superintendant of a bath establishment, is well aware of the great importance of constitutional treatment, both before and during the employment of any remedies applied directly to the surface of the body. Adopting the principles, that almost all cutaneous diseases are, in their commencement at least, of a more or less inflammatory nature, (a doctrine which was first insisted upon by the French physicians) he strongly inculcates the general propriety of antiphlogistic remedies at first.

In the acute forms, or when the eruption on the skin is accompanied with symptoms of constitutional excitement, the necessity of such a treatment must be too obvious to require any illustration. We need not allude to the more violent and severe exanthemata, as Variola, Rubeola, Scarlatina, &c. in proof of this assertion. In the milder diseases of Erythema, Roseola, Lichen, and Urticaria, the antiphlogistic regimen will be found to be equally salutary in practice. A single bleeding, followed by the use of a cooling purgative and of a light diet continued for a few days, forms by far the most successful treatment—in the early stages of such cases. Even in many of the more chronic forms of cutaneous disease, the advantages of a depletory regimen, at least as preliminary to the adoption of other means, have of late years been recognized by some of the most experienced physicians, both at home and abroad. The following observations of Rayer deserve to be generally known.

"I have derived the greatest benefit from venæsection in numerous chronic inflammations of the skin. Several practitioners restrict the measure of abstracting blood, in affections of the skin, to cases occurring in the strong and lusty, or in subjects of a sanguine or bilious constitution. For myself I declare that I have repeatedly had recourse to bloodletting, with the greatest advantage, even when it seemed to be contra-indicated by the general state of the patient, particularly in elderly persons suffering from sleeplessness caused by prurigos, lichens, and eczemas, that had resisted all other treatment. Avicenna long ago recommended the same practice.

In chronic states of inflammation of the skin, the blood is commonly buffy, even in the aged. This condition of the circulating fluid ought to be taken into account, as its appearance might incline us to recur to bloodletting oftener than is really necessary; and we must be very guarded not to change this state of the blood too rapidly by the repeated use of the lancet; the constitution of the patient would inevitably suffer: and farther, as I have frequently had occasion to observe, the blood becoming more and more serous, might, nevertheless, preserve its disposition to coagulate with the buffy coat, and thus lead to serious mistakes. In general, the bleedings ought not to be repeated but at somewhat distant intervals, once a month for instance, and at the periods of menstruation in females labouring under the skin complaints, which have been preceded by amenorrhœa or dysmenorrhœa." 66.

General as well as local bleedings by means of cupping, or of leeches applied near to the affected parts, have had the happiest effects in the treatment of many cases of Eczema, Impetigo, Psoriasis, Herpes and Prurigo. It is generally proper, to repeat the local abstraction of blood in young subjects, as often as the inflammation shews a disposition to extend, or is accompanied with much heat, or pain; and even in the case of elderly persons, the same treatment will be found to be the most simple and efficacious, provided their constitutions be moderately vigorous and healthy.

The use at the same time of the simple hot air or vapour bath, twice, or thrice a week, will often dissipate cutaneous diseases, which have obstinately resisted tedious courses of internal remedies.

A very prevalent error has long existed among medical men, and more especially among those of our own country, of supposing that most protracted cases of cutaneous disease, are always associated with, if not actually dependent upon, some disturbance of the chylopoietic viscera. Hence the almost constant resort to the use of purgatives, alteratives, stomachics, &c. on the very vague principle of correcting the state of the stomach and bowels. We have the authority of Rayer in stating that, in individuals attacked with chronic inflammations of the skin, the digestive apparatus is very commonly quite healthy; an observation, the truth of which the experience of most practical men, we are inclined to think, will confirm.

For many years after the appearance of Dr. Hamilton's book on Purgatives, almost every cutaneous disease was referred to disordered states of the bowels, and the treatment universally adopted, was the use of powerful and repeated doses of purging medicines.

This practice seems to have been as common in France, as in this country, and has there been known under the title of "Hamilton's Method," although, strange to say, there is no mention of cutaneous diseases, with the exception of one or two of the febrile exanthemata, in any part of his book. We must therefore wholly exculpate the admirable author himself, from the charge we have made. It is quite true, that the use of mild aperients or laxatives is almost always proper, if the bowels do not act regularly of themselves. Weak infusions of rhubarb, sweetened with manna, the different saline purgatives, as the sulphates of soda, of potash, and of magnesia, the tartrate of potash and soda, the sulphate of potash with sulphur, in the dose of one, or two drachms, in a large quantity of water, are perhaps, the most advisable. There is one medicine of this class, which has been long and most extensively used in almost every form of cutaneous disease, and is certainly of singular efficacy in many cases, probably from its exerting an effect upon the surface of the body, as well as upon the intestinal canal; we allude to sulphur. It may be administered either alone, or in combination with a saline aperient, or with an alkali, as the carbonate of soda, or of potash. Dr. Green is of opinion that it has been usually exhibited in too large doses. "In very small doses," he says; "and under circumstances favorable to its exhibition, it has in my hands proved a most valuable alterative, and most gentle, but efficient diaphoretic." In most cutaneous diseases, in which the internal use of sulphur is advisable, the simultaneous employment of it, in the form of vapour, or of lotion and ointment to the skin, will be found highly efficacious. The sulphurets of potash, soda, and lime are convenient forms for the preparation of artificial sulphureous waters, either for internal, or external administration. From half a pound to a pound of one of these sulphurets added to about thirty gallons of water, makes a "sulphureous water, or artificial Barrége bath;" and a "sulphureo-gelatinous bath" is prepared by dissolving from two to four ounces of the sulphuret in thirty gallons of water, and adding to the solution one or two pounds of isinglass previously dissolved in boiling water.

"This bath is preferable to the artificial Barrége's bath, as it is neither irritating, nor apt to occasion feverishness, which the common sulphureous water bath is.

A cheaper and not less efficacious gelatine may be procured by dissolving from ℔iss. to ℔ii. of parchment clippings in water, by long boiling, or by using a neat's or calf's feet for the purpose." 352.

The different preparations of the alkalis and of some of the earths have been generally esteemed highly useful in the treatment of cutaneous diseases. The liquor potassæ and lime-water were much valued by Dr. Willan, against the squamous diseases in particular; and the subcarbonates of soda and of potassa have always been justly praised, as admirable adjuvants to the action of sulphur. Ammonia and its carbonate may be frequently employed with the greatest advantage, especially in diseases presumed to be of a syphilitic nature, and whenever the general powers of the system are much enfeebled. In Prurigo, when it occurs in weakened constitutions, the internal use of ammonia alone, or combined with a bitter infusion, is sometimes of singular efficacy,* Alkaline baths and washes are easily prepared, and are remedies of great utility. From four to eight ounces of the subcarbonate of potass, added to thirty gallons of water, form a bath which is very useful in promoting desquamation from the skin, and in allaying Pruritus of obstinate Lepra and Psoriasis ; and a convenient lotion may be prepared, by adding from one to two drachms of liquor potassæ to an ounce of rose-water.

The acids are, perhaps, more frequently useful in cutaneous diseases than even the alkalis. The mineral acids, indeed, are among the safest and the most potent remedies which we possess against these maladies; and each of them appears to have properties peculiar to itself, and hence to be better adapted than the rest against certain orders of these. Thus, the diluted sulphuric acid, in doses of from ten drops to a drachm three times a day, has long been recommended in cases of ulcerated Eczema, and of the severe kinds of Lichen ; while the nitric acid has been supposed to be more useful in Impetigo and Pityriasis, not to mention its effects in the various forms of secondary syphilitic disease. The nitro-muriatic acid bath, prepared by adding three ounces of the nitric acid, and one ounce of the muriatic, to an ordinary tepid bath (which should then be about as sour as distilled vinegar), is of decided efficacy in numerous cases of obstinate skin disease. The acetic acid, diluted with water, is recommended by Hippocrates in various affections of the skin ; and, of late years, Mr. Wilkinson has again brought it into use against Lepra and Lichen. The hydrocyanic acid is extremely useful in allaying the pain and irritation of Impetigo, and in many scaly affections, attended with severe itching, or a wash, prepared by adding one or two drachms of it to six ounces of rose-water. The addition of some liquor plumbi, &c. may be made, according to circumstances.

So much for certain classes of medicinal agents. We shall now make a few practical remarks on certain remedies, whose mode of action is certainly not well understood, and which, although of singular efficacy in some of the most obstinate skin diseases, are, it must be confessed, very generally exhibited on mere empirical principles ; for example, mercury, iodine, and arsenic, &c. Well may Rayer say, that the preparations of mercury are universally employed in cutaneous affections, both internally and externally. From the common Tooth-gum of infants, to the frightful horrors of Lepra and Elephantiasis, every disease has been made to pass through the ordeal of mercurialization.

The preparations of this mineral, which have been most extensively used, are its proto-chloride and deuto-chloride (calomel and corrosive sublimate).

---

* The action or modus operandi of alkaline remedies in cutaneous disease, has not hitherto been examined with the attention which the subject deserves. One very important effect of their use is the increase in the flow, and the change in the quality of the urinary secretion, which is very generally more or less disturbed in all cases of protracted cutaneous disease. The morbid sympathies between the skin and the kidneys, and the therapeutic indications derivable from a knowledge of these sympathies, have often been noticed. It is well known that, in the calculous diathesis, the state of the skin is seldom quite healthy, and, on the other hand, that, in cutaneous diseases, the urine is generally very offensive.

It is almost unnecessary to enlarge upon their well-known therapeutic effects.

"I believe (says Rayer) myself to have been one of the first who observed that the white precipitate (calomel obtained by precipitation), mixed with lard in the proportion of a drachm to an ounce of grease, applied in frictions to the affected parts in the quantity of one, two, or three drachms daily, exerts a specific power over two forms of squamous inflammation, namely, lepra and psoriasis. I never saw these frictions followed by salivation, which is so frequent a consequence of the internal exhibition, even in very small doses, of calomel prepared in the ordinary way. In this respect, therefore, there is an actual difference between an ointment of precipitated calomel and the common blue or mercurial ointment, the action of which on the salivary glands is constantly the same. I have seen patients labouring under old and inveterate psoriasis use half a pound of precipitated calomel by way of friction, without their mouths becoming in the slightest degree affected, and obtain a perfect cure." 77.

*Sublimate baths*, prepared by dissolving from two to four drachms of the oxymuriate in thirty gallons of water, have been used with advantage at the Hôpital des Veneriens, at Paris, by M. Ricord and others. Rayer says:—"I have many times prescribed these baths, and have never seen salivation follow their use; but their good effects have also often appeared to me to be very questionable." We are not aware of their having been used in this country, nor is it likely that they ever will be so extensively. On the other hand, mercurial fumigations have been much more used in this country than on the Continent, in many parts of which, especially in Germany, they are as yet scarcely known.

"The mercurial preparation commonly used in the fumigating apparatus is the cinnabar or red-sulphuret; the proto-chloruret or calomel is also occasionally employed, but both of these are inferior in efficacy, I am inclined to believe, to the grey oxide; this has the great and obvious advantage, at all events, of not making patients cough, should a little of its vapour be breathed accidentally whilst they are shut up in the apparatus. The system is very readily and rapidly brought under the influence of mercury by means of the fumes of the grey oxide administered in a proper apparatus, even after all other modes of accomplishing this end have been tried and found unavailing." 304.

Within the last few years, the iodates or iodurets, and the cyanuret of mercury, have been strongly recommended in some cutaneous affections, especially when these occur in scrofulous constitutions. Iodine and its multifarious preparations and combinations have, it is well known, acquired so great renown in the cure of numerous forms of disease occurring in strumous patients, that "it has been energetically characterized as 'la bataille d'Austerlitz des Scrofules' by one of its most distinguished and able advocates in France, so completely does it rout them."

The only other heroic remedies to which we shall at present allude is arsenic. In obstinate cases of Lichen, Prurigo, and especially of Lepra, and other forms of scaly disease, arsenic has been found to exert the most powerfully curative effects. Certain chronic and obstinate forms of Eczema of the scrotum, margin of the anus and labia are, of all the vesicular inflammations, those in which arsenical medicines are most frequently and successfully employed. Cases of Lepra, which had lasted for fourteen, and even twenty years, and which had most obstinately resisted all other remedial means, have been cured by the administration of from three to twelve, or even twenty drops of Fowler's arsenical solution, taken three times daily. Its use must be continued for six or eight weeks, or even for three months, before we can hope to accomplish a radical and permanent cure of the evil, in such inveterate cases. True it is that, not unfrequently, it requires to be discontinued for a time, in consequence of the disagreeable effects which it is apt to induce. Pains in the chest and bowels, sickness, vomitings, fainting, and extreme anxiety at the præcordia, accompanied with quickening of the pulse, nervous tremblings, diarrhœa, oppression of the breathing, the sensation of tingling in various parts of the surface, which, at the same time, not unfrequently exhibits an erythematous or vesicular eruption, are very common effects of the internal use of arsenical preparations.

A very common result is, itching and pain of the eyes—the eyelids, especially the lower ones, becoming puffed, and surrounded by livid circles.* Whenever these symptoms occur, the remedy ought to be discontinued for a time, and resumed when they have ceased. M. Rayer says, that arsenical preparations have been known to induce paralysis and wasting of the genital organs. He alludes to three such cases, which have occurred in his own practice. In this respect it resembles iodine, which, it has been very generally believed, exerts in certain constitutions a very peculiar effect on the testis of the male, and on the mammæ of the female. A certain degree of analogy between the effects of arsenic and of mercury is also occasionally witnessed; both of these poisons induce, in certain states of the system, a vesicular eruption on the body, not to mention the resemblance between the constitutional disturbances which so frequently follow the use of them.

The action of iodine is, in many respects, very similar to that of mercury. They are used advantageously in the same classes of disease, and iodine, it has been frequently remarked, induces salivation like mercury. The circumstance of these three very active drugs appearing to agree, at least to a certain extent, in the effects which they produce on the system, may lead us probably to form a rational conjecture as to their modus operandi in disease, and thus to obtain some safe indications as to their use. According to our view of the subject, they are all excitants of the capillary vessels, as is shewn by the feverishness of the system, by the heat and irritation of the surface, and the other symptoms which not unfrequently follow their lengthened employment. It is in consequence of this action on the extreme vessels that they act so beneficially, not only in most chronic cutaneous diseases, but also in many others, which are accompanied with languor of the capillary circulation, and diminution or perversion of the nervous energies; as in Agues, Chronic Rheumatism, Paralysis, Neuralgia, &c. &c. The practical rule, therefore, which we should prescribe for their exhibition is simply this—that in all acute forms of cutaneous disease they should not be used; (an exception, to a certain extent, must, it is true, be made in favour of mercury;) and, on the other hand, that the cases which are most likely to be benefited by them, are those where the evil has existed long after all inflammatory symptoms have disappeared, and where it seems to be kept up

* The urine has been known to acquire a jaundiced appearance from the long use of arsenic.

by a morbid action of the skin itself, independent of any constitutional derangement of the health.

The admirable effects of arsenic in old cases of Lepra, Lichen, &c. and of mercury against all the syphiloid affections of the skin, have been long known and appreciated. Of late years, iodine and some of its preparations, especially the hydriodate of potash, have been found of very considerable efficacy in both these classes of diseases, as well as in Chronic Rheumatism, Paralysis, &c.

The few hints which we have thrown out may possibly be of use to the young practitioner in directing his practice in cases, which hitherto he has treated empirically, and, as it were, with a mere random aim.

Having thus briefly alluded to the principles which ought to direct our treatment of cutaneous diseases, and specified the most potent and generally useful of the remedies which the healing art affords, we shall now proceed to take a rapid survey of some of the most interesting orders of this class of maladies, the consideration of which was omitted in our previous review of Rayer's work, commenting on their most remarkable features, and more especially on their therapeutic treatment.

The classification which we shall follow is that adopted, with very slight modifications, by Dr. Green from his French contemporary; for in the arrangement, as well as in the descriptions of individual diseases, Dr. G. has drawn largely from the ample stores of Rayer's Treatise, the contents indeed of which constitute a complete encyclopædiacal history of whatever is known of Dermatology.

The following is the tabular view which Dr. G. has given.

| | |
|---|---|
| Forms of Inflammation of the Skin, and Diseases which appear under these severally. | EXANTHEMATA: Erythema, Erysipelas, Roseola, Rubeola, Scarlatina, Urticaria.<br>VESICULÆ: Miliaria, Herpes, Scabies, Eczema.<br>BULLÆ. Pemphigus, Rupia.<br>PUSTULÆ: Variola, (including Varicella,) Vaccina, Ecthyma, Impetigo, Porrigo, Acne, Mentagra.<br>PAPULÆ: Strophulus, Lichen, Prurigo.<br>SQUAMÆ: Pityriasis, Psoriasis, Lepra.<br>TUBERCULA: Lupus, Elephantiasis Græca, Cancer, Molluscum, Frambœsia.<br>FURUNCULI: Furunculus, Anthrax, Pustula Maligna. |
| Diseases which appear with the elementary characters of almost all of the above orders. | Syphilis. |
| Diseases which are severally types of new and additional orders. | Pellagra.<br>Purpura.<br>Elephantiasis Arabica.<br>Cheloidea, (Keloide, Alib.) |
| Original or accidental unusual states of the skin, not referable to Inflammation. | ACHROA: Leucopathia, (Albinismus,) Vitiligo.<br>DISCHROA (MACULÆ, Willan): Lentigo, Ephelis, Chloasma, Nævus. |

| | |
|---|---|
| Diseases of the appendages of the skin; more properly of the parts which secrete and support these." | Epidermis—Ichthyosis. Ungues—Onychia. Pili—Plica. |

As we have already expressed our opinions in a former number of the merits and demerits of Rayer's classification, we deem it unnecessary to enlarge upon this subject. No one now thinks of arranging with Willan Erysipelas among the bullæ, or Purpura among the exanthemata; and certainly some of the diseases grouped together under Willan's order of Tubercula are most dissimilar in all their essential characters. The close affinity between the genuine Variola, Varicella, and Vaccina, requires that these diseases should be brought together, and not separated, by one being placed among the pustulæ, and the other two among the vesiculæ. (In our former review we suggested the propriety of establishing a vesiculo-pustular order of skin-diseases, in which all the disputed members—we mean such diseases as are truly vesicular at their commencement, and which become pustular only during their progress, might be conveniently inserted. Our subsequent reflection has confirmed our opinion as to the propriety of such an arrangement.)—*Rev.*

Rayer, although sensible of these and some other defects of Willan's classification, bears a generous testimony to the surpassing merits of the English physician. The passage is altogether so honorable alike to the eulogized and the eulogist, and conveys so salutary an example for imitation to authors of both countries, that we cannot deny ourselves the pleasure of transferring it entire to our pages.

"The great characteristics of Willan's writings are the impress they bear of the scientific spirit that guided him in his researches; the great precision, and the purity of his descriptions; the particular pains he takes to select well, and to use judiciously, his technical expressions; lastly, the taste and the sound judgment he displays in his interpretation of the ancients. If there be ought with which he is chargeable in the way of omission, it is with having paid too little attention to the relations of the diseases of the skin to the state of the constitution, to anterior diseases, and to those affections which are attributed to their repercussion. His therapeia is in general active; his practice, his writings, and the works which have issued from his school, contributed powerfully to extend the free use of purgatives, and the internal employment of a variety of powerful medicines, such as the tincture of cantharides, the mineral acids, and the preparations both of antimony and arsenic in the treatment of cutaneous affections." *Introd.* lii.

The Exanthemata are characterised by a diffuse inflammation of the skin in small or large patches, terminating in a separation of the epidermis in scales, which are sometimes so minute as to be almost imperceptible. The chief danger of this class of cutaneous diseases is, the occasional complication of the evil extending to the pulmonary, or gastro-enteric mucous membrane. More rarely there is a tendency to cephalic disturbance. In all cases, therefore, of serious exanthematous disease, it is very necessary to ascertain the state of these important organs.

"In the exanthemata, the limits of the dermis and vascular rete are much more easily demonstrated than on the healthy skin; a simple incision through the substance of the skin is enough to exhibit these two layers; it is then, in truth, as M. Gendrin well observes, if at any time, that we must be induced to conclude that the vascular reticulation and the dermis form two distinct and superposed membranes. When the inflammation has run high, the vascular rete is of a red, and even of a brownish colour, as in erythema nodosum, rubeola nigra, and gangrenous erysipelas ; a certain quantity of blood then appears to be extravasated into the tissue of the skin." 100.

The description of Erythema and its varieties given by Rayer is full of interest and instruction.

The evanescent forms of the disease ally it very closely to Roseola; and its well-known affinity to Erysipelas needs not to be pointed out.

Under the head of Erythema, Dr. Green has noticed the *novel* epidemic disease to which the French authors have given the name of *Acrodynia*. It was first observed in June 1828 at the Hospice de Maria Therese at Paris, and soon afterwards in various parts of the city. The troops in the different barracks suffered severely from it: in one, 560 out of 700 soldiers were attacked with it. During the following Winter it abated; but returned in the Spring; and finally appeared to become quite extinct in the Winter of 1829-30. The symptoms of Acrodynia were as follow. Generally after some feverish disturbance of the system, "the skin of the palms of the hands and soles of the feet presented, often from the invasion of the disease, a red colour which frequently extended along their edges to other parts of the body, to the legs more especially, in the shape of patches of various shades of red, sometimes very similar to ecchymoses. Pretty frequently, too, the skin assumed a blackish or brown appearance as if it had been rubbed over with soot, this happened more particularly on the abdomen, neck, and about the bends of the joints; it very rarely happened that this discoloration extended to the face."

In many cases, numerous pustules, phlyctenæ, and even boils, appeared on the hands and feet, and sometimes the corpus mucosum was laid completely bare in various parts, when the process of desquamation, assisted by the profuse local sweats, commenced. The patient was much distressed with a sense of cold followed by one of burning heat, of numbness, tingling, or even sharp shooting pains in the hands and feet, more especially in the latter, extending up the limbs, but always more severe at the extremities than elsewhere, so that the slightest pressure on them caused severe pain. In a few cases, these parts were affected with complete paralysis, accompanied with contraction and wasting of the limbs. The duration of this singular disease varied from a few weeks to several months. It rarely proved fatal, except among the old and infirm. Its probable cause was very obscure. Some attributed it to the use of unwholesome food, others to a peculiar unhealthiness of the atmosphere; but it was observed to prevail in the cleanest and best supplied barracks, more than in the hovels of the ill-fed poor. It did not appear to be contagious.

"The disease was viewed variously as rheumatic in its nature, as an inflammation of the skin and mucous membranes, as the effect of a peculiar lesion of the nervous system, especially of the spinal marrow, lastly, as a new disease.

*Diagaosis.* Pellagra of all known diseases is that with which acrodynia has the greatest analogy; both are characterized by a triple manifestation of cutaneous symptoms, of gastro-intestinal symptoms, and of nervous symptoms, the similarity of which is certainly striking. (vide *Pellagra.*) In several epidemics attributed to indifferent qualities of the cerealia, to spurred rye, &c., the greater number of the symptoms of acrodynia will also be found noted, such as tinglings and numbness of the feet and hands, amounting sometimes to paralysis, contraction of the fingers, cramps of the legs, swelling of the feet, phlyctenæ on these parts, &c. These epidemics, like that of Paris of 1828, also occurred in cold and moist summers." 1178.

The treatment of Acrodynia was certainly very unsatisfactory; for generally, in spite of all kinds of remedies, its progress was very slow to recovery. Reference is made by Rayer to all the best memoirs on this disease; and in the excellent description which he has given of that pest of the fair plains of Italy, the Pellagra, he has pointed out the analogies which may be traced between it and the Parisian epidemic.

In some parts of Spain, and especially in the Asturias, the "Mal de Rosa," a cognate disease to the Pellagra, is not unfrequently prevalent.

The treatment of Erythema, at least of its more common varieties, is sufficiently well understood by every well-educated medical man. After Erythema, we may introduce Roseola; the two diseases, in several of their forms, meeting and running into each other. "Let but erythema," says Rayer, "become somewhat more general than wont, and its spots appear a little more prominent, or the patches of roseola, from some unusual violence of the accompanying inflammation, become particularly prominent, and the appearances of these two exanthemata are the same."

The affinity between some of the forms of Roseola and Urticaria is very striking. Both are very often induced by slight derangements of the digestive system, as by certain articles of food, which disagree with the stomach. The diagnosis of Roseola is chiefly important, during the prevalence of Measles, or of Scarlet Fever, as the error of mistaking it for either of these diseases, has been often committed, and has thus led to the belief that a second attack of Measles and Scarlatina is not unfrequent.

The exciting causes of this exanthem are various. The heat of Summer weather, the irritation of dentition, the inoculation of the variolous and vaccine lymphs, and indeed the feverish disturbance of the system, from whatever cause, very often induce an eruption of roseolous inflammation on different parts of the surface. A roseolous or erythematous form of efflorescence is sometimes connected with attacks of Gout and Acute Rheumatism. Some of the German authors have described this variety under the title of Peliosis Rheumatica.

It appears to be unusually common at Wurzburg, where rheumatic affections are almost endemic and frequently fatal, from their being complicated with Miliaria.

In the 33rd vol. of the Edin. Med. and Surg. Journal, Dr. Cock has minutely described,* an "Epidemic Eruptive Rheumatic Fever" which he saw in the West Indies.

During the prevalence of the Asiatic Cholera in France and England, two years ago, a Roseolous Eruption was observed in several of the patients, when the period of re-action had set in. In some cases, it resembled the efflorescence of Scarlatina, in others, it had more of the appearance of the rash of Rubeola, or of Urticaria.

In the description given by Rayer of Erysipelas he alludes to its supposed occasional contagiousness.

He is rather inclined to the negative opinion, and attributes the spreading of the disease in certain seasons and places to epidemic influences, or states of the atmosphere, the nature of which is not yet understood. Dr. Tweedie on the other hand is quite convinced of the occasional contagiousness of Erysipelas: he says

"When we find persons who, after becoming infected apparently from attendance on erysipelatous patients, remove as soon as they become ill to another residence at some distance, and communicate the disease to the family, the irresistible conclusion is, that erysipelas in such cases has been communicated by contagion." 110.

The blood in cases of Erysipelas is sometimes quite as pertinaciously buffy, as in acute Rheumatism. The desquamation of the cuticle, after severe erysipelatous inflammation, takes place generally in large flakes. Mr. Wilson, the late Lecturer on Anatomy in London used to mention the case of a patient who was subject to attacks of Erysipelas, at the end of which the cuticle of the hands and feet was detached in the form of a glove. The characters of erysipelatous inflammation are considerably modified by the peculiar structure of the parts affected. When it affects the hairy scalp, it is almost always of that kind, which has been called Phlegmonous Erysipelas, in which the subcutaneous cellular texture, as well as the skin itself, is involved, and in which the attack is often of alarming severity.

"If this disease, be left to itself, shivering fits at irregular intervals supervene, and the patient falls into a state of coma. The inflamed skin becomes thin in parts, bursts, and gives vent to a quantity of pus and of shreds of cellular membrane and of the occipito-frontal aponeurosis in a gangrenous state. The scalp is almost never stricken with gangrene; being, according to the judicious remark of M. Dupuytren, furnished with blood vessels which are independent of those that are distributed to the pericranial cellular substance. On the following days new openings are formed in the most depending parts near the centre of the erysipelas, and additional shreds of cellular membrane and of aponeurosis are detached; the discharge is abundant and offensive, the bones of the cranium are sometimes exposed, and if the disease be not checked in its progress, dilirium, diarrhœa, and various other serious symptoms give warning of the approach of death." 127.

The Erysipelas of the umbilical region and genital organs, principally observed among the new-born infants in hospitals, has a marked tendency to terminate in gangrene. It is frequently complicated with peritoneal inflammation, and with umbilical phlebitis. The causes of this species are not well understood. Injury of the umbilical cord, bad food, unwholesome air, have appeared to induce it. Erysipelas of the scrotum and prepuce occurring in elderly persons often ends in gangrene. Dr. Tweedie observes—

"Of protracted cases of malignant fever especially, it (Erysipelas) is a frequent consequence; and we have occasionally observed that the inflammation commenced and was confined entirely to the throat: more generally, however, the inflammation, after beginning in the throat, has spread from the mouth to the cheek and face, or through the nostrils to the nose, and thus erysipelas has been propagated to the face and head. When this form of pharyngeal inflammation is confined to the throat, it appears to us to be in some measure allied to the *Diphtherite*, of which Bretonneau has given an excellent description. There is, however, no pellicular or membranous exudation, which forms the characteristic distinction of diphtherite—indeed, in several fatal cases, in which this pharyngeal inflammation was combined with erysipelas of the face and head, we have found scarcely any traces of the previous existence of inflammation." 108.

With respect to the immediate etiology or proximate cause of Erysipelas, M. Ribes has lately announced, as the result of his pathological examinations, that the internal tunic of the veins and arteries of the integuments is inflamed, and that the disease principally attacks the small cutaneous veins, the small arteries being less affected. Probably Rayer is more correct in his suspicions. Describing a case of Erysipelas of the face, which proved fatal, he states

"The subcutaneous and intermuscular cellular tissue of the face was infiltrated by a yellowish sero-purulent fluid; small abscesses, containing laudable pus, existed in front of, and behind, the orbiculares palpebrarum muscles, among the cellular tissue of the orbit, and extended towards the temporal fossæ; the cellular substance of the scalp itself was also infiltrated. The parietes of the veins of the face and neck, although lying bathed in pus, showed no trace of inflammation. Many of these minute vessels contained purulent serum similar to that effused into the inflamed cellular membrane. The minute arteries were healthy. I have also found pus in the lymphatic vessels of a lower limb attacked with phlegmonous erysipelas, without any visible alteration of the parietes of the blood-vessels. In fine, I have met with true inflammation of the principal veins in limbs that were the seat of phlegmonous erysipelas or of simple phlegmon, and as a consequence of paronychia or of amputation. If I may be allowed to draw a conclusion from my own experience, I should say, that phlebitis complicates inflammation of the cellular tissue more frequently than that of the skin." 129.

As to the prognosis we may state generally, that deep and extensive Erysipelas of the limbs, that diffuse and wandering Erysipelas, and that the sudden and spontaneous disappearance of the cutaneous affection, occurring during the course of chronic diseases, are always of very serious import. Whenever there is reason to believe that the disease is complicated with Phlebitis, it is almost always fatal. The treatment of Erysipelas inculcated by M. Rayer is most praiseworthy.

It may be styled truly "British," for its activity and decision. Blood-letting is by far the most powerful remedy in all cases of the acute form, when the constitutional symptoms run high. While he admits the utility of the tartar-emetic medication, he distinctly affirms the superiority of blood-letting in the majority of cases. The precept of the Scotch maxim, "baith's best," will be followed by the judicious practitioner. The application of mercurial ointment to the inflamed surface, is, according to Rayer's experience, utterly useless. Simple lard is quite as effectual. Flour and other dry powders, so generally applied some years ago in Erysipelas, are not to be recommended. The results of several trials by M. R. to stop the progress of Erysipelas by the superficial cauterization with the nitrate of silver, have not been at all uniformly favorable. This remedy has been in our practice of great utility in several of the forms of Erysipelas; not only in the more common form, where the disease is limited to the more dermoid texture, but also in that more deep-seated form where the subjacent parts also are involved. We are confident that in many cases, the termination of the latter form in suppuration and gangrene has been prevented by the free application of the nitrate to the affected integuments. In severe cases, the caustic may be applied to the entire surface of an extremity. This remedy is vastly preferable to blistering the inflamed parts, as recommended by Dupuytren and other French surgeons. Intermittent Erysipelas is a very rare disease. Rayer has seen it in the face, associated with Neuralgia of the affected part. Both diseases were cured at the same time by the quinine. Compression is proper only in Œdematous Erysipelas, or when the severity of the inflammatory action has been already subdued. The admirable effects of free incisions in severe cases of Phlegmonous Erysipelas affecting the scalp and limbs, cannot be gainsayed by any practical surgeon.

"The more indolent and lingering forms of erysipelas, especially of the lower limbs, attended with much œdematous swelling, are always greatly benefited by the hot air, and still more by the sulphur fume bath. The disposition to attacks of this kind exhibited by some individuals, may be got rid of entirely by the occasional use of this means."—*Green,* 40.

*Urticaria.* Rayer has shewn great judgment in consolidating the six species of Willan, viz. U. febrilis, evanida, perstans, conferta, subcutanea, and tuberosa, into two—the acute and chronic, according to the circumstances of the case. Urticaria is a troublesome, but seldom or never a dangerous disease. It has been known to be associated with Agues, and then it has appeared to be epidemical. It is one of the cutaneous affections which belong to the class of "rheumatic eruptive fevers."

In the treatment of acute Urticaria, it may be asserted positively that, whenever it does not promptly yield to an emetic or cooling purgative, the detraction of blood will be found most serviceable. In the more severe form, which has been denominated U. tuberosa, from the size, depth, and hardness of the wheals, the blood, Rayer says, is usually in a buffy state. The chronic forms require the persevering use of mild alteratives and tonics. The mineral acids are among the best. Before leaving the exanthemata, we shall briefly allude to two topics in the history of Scarlatina.

It is well known that Hahnemann, several years ago, announced that belladonna, taken internally, was a powerful preservative against the attacks of this disease. He has succeeded in convincing very many of his own countrymen, and also a few of the French doctors, of the truth of this grand discovery! A host of *well-authenticated* and *most indisputable* cases have been published in consequence; but all this evidence does not appear to have satisfied the incredulous minds of British physicians. Rayer contents himself with merely chronicling the opinions of others, and does not state his own. From this, we may fairly infer that he is still a sceptic. Dr. Green is more easily satisfied; but we fear that his authority will have little weight, especially as he confesses that he has had only one opportunity of witnessing the prophylactic powers of belladonna. "Among the children of a boarding-school, where Scarlatina had broken out, four, to whom the medicine was administered, escaped the disease entirely." Most conclusive evidence! Dr. G. has followed his Continental authorities too implicitly on this occasion.* The formula recommended is, to dissolve 6 or 10 grains of the extract in an ounce of water, and to give from 5 to 20 drops of this solution three or four times a day. The modus operandi is, *as a matter of course,* beautifully illustrative of homœopathic principles! It excites a disease in all respects similar to Scarlatina. The following case, from Rayer, may be adduced as an example of its effects on the skin.

* Dr. Tweedie, in his able description of Scarlatina, in the Cyclopædia, is less

" M. N. forty-six years of age, having at four o'clock in the morning taken forty-four grains of belladonna, was seized about an hour afterwards with supra-orbitary head-ache of the most violent description, and excessive redness of the skin, which first appeared about the eyes and face, and then extended to the whole of the integuments. Within a few minutes the entire surface of the body presented a uniform red tint, exactly similar to that observed in scarlatina; the throat of the patient was also intensely red, and affected with heat, which seemed to be propagated through the whole course of the alimentary canal. Another remarkable symptom was this: that the urinary passages, and especially the neck of the bladder, became extremely painful. The patient, in the midst of his delirium, was perpetually asking for the pot de chambre, and it was with difficulty that he succeeded each time in passing a few drops of red and bloody urine. All these distressing symptoms were relieved by a copious bleeding, demulcent drinks, soothing glysters, and the application of leeches." 205.

The other topic deserving our attention is the occurrence of dropsical affections, so frequent during the decline of Scarlet Fever. Rayer is inclined to agree with many English physicians, in regarding these attacks as generally inflammatory in their origin, and, therefore, as requiring the use of antiphlogistic remedies for their cure. The pathological history of this variety of dropsy has not, hitherto, been well ascertained.

" Blackall and, more recently, M. Peischer, have shown that the urine was often albuminous during the continuance of the anasarca; and there is enough in these scattered remarks, when united, to make us enquire whether this species of anasarca be not really a variety of the dropsical affection lately made known by Dr. Bright, which Drs. Gregory and Christison have done so much to illustrate by new facts, and in the investigation of which I have myself spent some time.* In fact, like the disease described by Dr. Bright, the anasarca of scarlatina is almost always produced by exposure to cold and moisture. On the invasion of both diseases, a particular change in the qualities of the urine is often observed; this fluid becomes brown in colour, albuminous, and loaded with cruor. Both are very formidable affections; they both, occasionally, end in hydrothorax, and in hydrocephalus, and are very different from those passing dropsies proceeding from some obstruction to the current of the blood, the mechanism of which M. Bouillaud has so well explained. I have never had an opportunity of examining the organs, and particularly the kidneys, of an individual who has died from anasarca following scarlet-fever, although I have very frequently had such opportunities in reference to the disease described by Dr. Bright. I observe no dissections of such cases among all those published by Dr. Gregory; but there is during life, so perfect an identity in the phenomena of these two diseases, that post-mortem examination would, most probably, show them to be of the same nature." 171.

The most approved treatment consists in the use of bloodletting, if the patient's constitution warrants such a measure, and of repeated warm baths, and in the administration of calomel, of the acetate of potash, and other diuretics. Dr. Green says—

" Should symptoms of anasarca appear during convalescence, their progress may be almost certainly arrested by the use of the vapour-bath. Besides being

---

believing :—" We certainly (says he) profess ourselves sceptical as to the powers (preservative) ascribed to belladonna."

* Tissot, De l'Hydropisie produite par l'Affection granuleuse des Reins. Paris, 1833, 4to.

the most effectual and speedy means of checking this frequent and unpleasant attendant upon convalescence from scarlatina, the vapour-bath has the advantage of not only not retarding the patient's ultimate recovery, as all the other curative measures usually enforced necessarily do, but even of accelerating it." 61.

*Bullæ.* Rayer in the present edition of this work, appears to have followed the advice which we gave him last year, in reference to the impropriety of transferring Zona from the order of Vesiculæ to that of the Bullæ. The passage, in our Number for October last runs thus :—

"With respect to Zona, we have no doubt in saying that Rayer is wrong, when he places it among the bullæ : it is most decidedly a vesiculous disease ; and a critical reader cannot fail to be surprised at finding the author himself admitting that 'Zona, indeed, really differs from the Herpes Phlyctenoides only in the singular form it assumes,' as its very name imports ; for, whatever be its seat, 'Zona shews itself under the form of a semicircular band, of more or less extent, covering part of the trunk, or of a limb.' Perhaps it would be strictly more correct to designate Rupia and Zona as bullo-vesiculous, than as simply bullous or vesiculous diseases."

The following passage, from Rayer, expresses the same sentiments.

"When herpes zoster or zona, has attained its complete development, it very frequently appears with the characters of a *vesiculo-bullous* inflammation, which seems to form the link of connexion between bullous and vesicular diseases. Willan assigned this disease its true place among the *herpetes*, and I committed an error when I removed it from that group in the first edition of this work." 206.

If Rayer and other dermatologists would apply to other portions of their classification, the principle which we have urged, of applying compound epithets, as vesiculo-bullous, vesiculo-pustulous, to certain groupes of cutaneous diseases, instead of separating the members of these groupes from one another, and placing them in different orders, we are satisfied that their arrangement, would be not only more natural, but also more practically useful to the student of medicine. Perhaps the only genuine bullous disease is Pemphigus, including the Pompholix of Willan. The other genus Rupia, admitted by Rayer, has a very questionable right to be classified with it. The eruption of the former disease has more of the vesiculous or even of the pustulous, than of the bullous characters, for the bullæ are described as being usually " small, flattened, and as containing a fluid, which if not sanious, or purulent from the commencement, very quickly becomes so." The marked resemblance between many of its features, and those of Ecthyma Cachecticum, has been noticed by most authors, and indeed Mr. Plumbe has classified them together. The remarks of Rayer on this topic will be read with advantage.

"Rupia differs from ecthyma in its primary form, which is bullous, whilst that of ecthyma is pustular ; the base of the pustules of ecthyma is much inflamed, and the scabs with which they become covered at a later stage of their progress, are hard, and, as it were set, or incased within the substance of the skin ; the circumference of the bullæ of rupia does not present the same degree of inflammation, and their incrustations are much broader, more prominent, and less adherent than those of ecthyma. It must be allowed, however, that the bullæ of rupia become purulent very quickly, and that occasionally the diagnosis is rendered so much the more difficult, as the two eruptions are met with at one time in the same individual. Nevertheless, the prominent incrustations, and the deep, and often intractable ulcers of rupia are very different from the impacted scabs and slighter sores of ecthyma." 234.

Both diseases indicate a cachectic state of the constitution, and require for their successful treatment, the use of a generous diet, tonics, alteratives, &c. and residence in a pure air. The sulphur fume bath, or the artificial sulphureous water bath, made by adding three or four ounces of the sulphuret of potash to a common tepid bath will be found of great service, by stimulating the cutaneous surface, and improving the general health. The local treatment recommended by Rayer is

"After the fall of the scabs in rupia simplex and rupia prominens, the ulcerated skin is to be bathed with decoction of althea if it be painful; but if the inflammation appear indolent and below the pitch requisite for the production of a new epidermis or the formation of a cicatrix, it may be stimulated with a wash of wine and water, or a weak solution of cream of tartar. I have been in the habit of ordering the sores of rupia to be dusted with cream of tartar, and of all the topical applications I have tried, this is the one that seemed to me to answer best." 234.

*Vesiculæ.* The only change introduced by Rayer into his classification of vesiculous eruptions, since the appearance of the first edition of his work, is the unnecessary admission of the new genus "Hydrargyria" which had previously been arranged as the "Eczema Mercuriale." Dr. Green has done right not to follow him in this particular; but he has not shewn the same sagacity, when he makes this observation that "Herpes and Eczema are essentially chronic in their nature." His own description of acute Eczema falsifies this strange assertion, and with respect to Herpes, one of the very earliest remarks of his French preceptor in the account of Zona is "I have never met with it as a chronic disease," and he adds that the blood has been buffy in almost all the patients affected with Zona, for whom he had occasion to prescribe venæsection.

The entire description of this variety of Herpes given by Rayer is most minutely correct, both as regards its appearances and treatment. He alludes to the effect of the application of the nitrate of silver to the affected parts in the following terms:

"I find now, 1st. that if, after having opened the vesicles, or removed the cuticle by excision, their bottoms be very lightly touched with the nitrate of silver, in the same way as is often done in aphthæ, the duration of shingles is abridged, and that, on the contrary, it is prolonged, if the escharotic be too freely and carelessly applied; 2nd, that the vesicles, when properly cauterized, are more rarely followed by excoriations, or by eschars, than those that are left to themselves, especially in elderly persons, and when they are situated upon the posterior parts of the body; 3rd, that this system, which may be omitted in slight and distinct cases of shingles, ought to be put in force whenever there seems reason to apprehend excoriations or eschars in any of the groups on the body, face, &c.; 4th, that in touching slightly the red patches which precede the eruption of the vesicles, and especially those which appear subsequently to the first crop of clusters, their farther development is almost always arrested, but that the acute pain which accompanies them is not modified or abated." 260.

Being on the subject of Herpes, we may briefly allude to a mistake, which is still occasionally committed by young practitioners, and which has arisen from the confusion in the nomenclature of diseases; we mean, that of supposing that the Herpes Circinatus, or Vesicular Ringworm is ever contagious. Had the appellation of "Ringworm," not been applied to two essentially different diseases, viz. this variety of Herpes, and the intractable Porrigo Scutulata, we should never have heard of this error. As Rayer rather quaintly says, the former disease "cannot be and never is propagated by inoculation." It is not unfrequent to observe several cases of H. Circinatus occurring simultaneously in schools, &c. but this arises merely from all the children being exposed to the same morbific causes.

The treatment of Herpes Circinatus is very simple, and is quite similar to what is adapted to the other species of the disease. "Bateman," says Rayer, "recommends washes of sulphate of zinc, or borax, or alum, to soothe the itching that attends the formation of the vesicles in herpes circinatus. The application of a linen rag dipped in plain cold water, and frequently renewed, answers the same end completely. Alkaline baths and saline lotions have likewise been successfully employed in this complaint. I am in the habit of recurring to slight cauterization with the nitrate of silver, with very good effects. In a word, the mode of treating herpes *circinatus* does not differ from that which has been recommended in herpes *phlyctenodes.*" 272.

The next vesicular disease after Herpes, is the equally common one of Eczema. It is highly important for the medical practitioner to be well acquainted with the varying appearances of this eruption; not indeed, that he is to expect to find the features of every case to correspond precisely with the descriptions of any authors, but that he may be able to recognize the original and essential characters of the disease, although modified by constitution and other circumstances, or associated and complicated with other cutaneous affections. The history of eczema affords another apt illustration of the remark which we have previously made, that a great number of skin diseases have a truly compound character, and exhibit transitional appearances during their progress. Willan and Bateman acknowledged the very close affinity between Eczema and Impetigo, by admitting among the species of the former disease, the 'Eczema Impetiginodes.' On this subject, Rayer very accurateiy observes,

"Eczema and impetigo have between them many strong points of resemblance, as well in reference to the parts of the body most commonly affected, as perhaps in regard to the constituent element of the skin, the follicles, in which they are both evolved; it is not, therefore, uncommon to meet in the same individual with impetigo affecting one quarter, and eczema developed in another. It often happens also that we find a mixture of the vesicles of eczema and of the pustules of impetigo covering surfaces of the integuments of greater or less extent, and still more frequently do we find the vesicles of eczema becoming purulent and giving occasion to an anomalous variety of the disease which has been described by Willan, under the title of eczema *impetiginodes*. When this variety makes its attack in an acute form, the tension, heat and redness are considerable; it is not now mere tingling and itching that are complained of, but shooting and violent smarting pain. The vesicles now pass rapidly into the purulent state; the cuticle, raised in large flaps, is impregnated with the fluid effused, and acquires the appearance of greenish coloured laminated scabs, which being before long detached, a surface is exposed of as bright a red as carmine. When the eruption is considerable, the ichorous fluid secreted is so profuse that dressings of every kind, and even the bed-clothes and bedding become drenched with it; the smell of this matter too is as offensive as possible; it is faint and sickly, and something like that which a large burned surface in a state of suppuration diffuses." 285.

All this is strictly correct; but we think that Rayer, and following his authority, Dr. Green, have most unnecessarily transferred that species of Impetigo, which is almost peculiar to infants and children, and which has been so long known by the name of Crusta Lactea, (injudiciously arranged in the genus Porrigo by Willan) among the Eczemata. It is certainly rather a pustular, than a vesicular disease.*

---

* We regret to find that Dr. A. T. Thomson, in the article Porrigo, in the Cyclopedia, has not only most annoyingly (contrary too to the express opinion of Bateman) adhered to the old arrangement of the Crusta Lactea in this genus, but has injudiciously added to the confusion which had already existed in the

Eczema is very apt, under certain circumstances, to become chronic ; and then its distinctive characters are apt to be much changed from their original appearances.

"When the inflammation declines in severity, *chronic* eczema assumes another character. After the lapse of a longer or shorter interval, the vesicular, or vesiculo-pustular eruptions become rarer, and even end by not appearing at all; the scabs, at first moist and thick, and reproduced as soon as detached, grow thinner and thinner, drier, and more adherent to the skin, which at length appears covered by small yellowish coloured scabs,—the *dartre squameuse ou furfuracée* of some authors—among which, several bloody incrustations, the consequence of the excoriations caused by the nails of the patient, may be detected. The serous exudation is replaced by a simple epidermic exfoliation, to a greater or smaller amount. The more severe these eczemas have been, and the longer they have continued in this state, the longer is their complete disappearance expected, even after amendment has begun, and longer still are certain sequelæ, by which the previous existence of the disease may be certainly recognized, of being completely obliterated." 287.

Hence cases of chronic Eczema are not unfrequently mistaken for Psoriasis.

The treatment of the active or acute forms of Eczema is simple and usually quite successful.

The antiphlogistic regimen, general bleeding in some cases, cooling purgatives, the vegetable and mineral acids, a light mild diet, tepid baths, and the local application of cold water to the affected surface, will almost always lead the malady to a fortunate issue.

The chronic forms of the disease are, on the other hand, most distressingly refractory. Dr. Green states—

"When eczema appears with decidedly chronic symptoms, the sulphuric or nitric acid in conjunction with general dietetic measures will be found a medicine of great value. Any of the neutral salts in gently purgative doses continued for some length of time, frequently proves of signal service in these cases. The alkalis as well as the acids also deserve a trial; the liquor potassæ in doses of ten drops in barley water, twice a day, and gradually increased, has sometimes the best effects in modifying eczemas of old standing. When the pruritus is very troublesome, an alkaline bath taken in the evening will often be found to allay this distressing symptom greatly, and procure the patient a good night. The sulphureous water bath has frequently the same effects, and seems farther, in many old cases, to modify the eruption favourably. When the disease is indolent, the more powerful stimulus of the vapour bath or douche may be tried, often with the happiest effects; but the action of this bath requires to be closely watched, as eczema will not bear to be tampered with in any of its forms, and the application of stimulating remedies of all kinds is apt to be followed by an aggravation in the symptoms of the disease. For this reason it is, that the hot air and sulphur fume bath are seldom applicable in cases of eczema. They can only by possibility do good when the disease is in an indolent state, and when the system has been prepared for their action by previous depletion and abstinence. Then, and especially among the aged, I have known them of service in this disease after all other means had been tried and proved unavailing.

During the course of the whole of the varieties of chronic as well as of acute, local as well as general, eczema, a wash of the liquor plumbi sub-acetatis and emulsion of bitter almonds, or of the diluted hydrocyanic acid, may be freely used with a view to allay pruritus." 81.

---

history of Porrigo by introducing a most unnecessary arrangement of his own. He speaks of the true Porrigo, (including P. Larvalis, Lupinosa, and Favosa,) the eczematous Porrigo, (P. Furfurans,) and the anomalous Porrigo, [of which he makes two species, P. Scutulata and P. Decalvans!]

Some obstinate cases have yielded to the internal use of arsenic, after all other remedies had failed.

The only notice which we can afford to take of the next disease in the vesicular catalogue, Scabies, or the Itch, is to recommend a formula for an ointment, which was communicated to Dr. Green by Sir William Russell, as of great efficiency in India.

" ℞. Sulph. loti ℥ss. sodæ boracis, ammon. muriat. āā ʒss. hydrar. præcip. albi, ℈i. ungt. simpl. ℥i. to which ʒij. or ʒiij. of ol. terebinth. may be added or not at pleasure." 67.

There can be no doubt, that the only certain method of curing the disease, which King Jamie held crowned heads alone were worthy of having, [so supreme, according to his notions, was the pleasure of scratching,] is by the free local application of sulphur in the form of ointment. Sulphureous washes, baths, and fumigations cannot be depended upon.

The next order of the cutaneous diseases, is the Pustulous ; in which is included some of the most common and interesting cutaneous diseases, as Variola, the different forms of Varicella, Vaccinia, and also its varieties and modifications. At present we shall not enter on the consideration of any of the important questions connected with this most important groupe of cognate diseases, as it is our intention, if we have room, to make some remarks upon them in a subsequent part of this number, when treating of vaccination. Acné, including the Rosacea, or Couperose of the French authors, and Sycosis, or Mentagra, are, although obstinate, never dangerous affections. We must refer our readers to Rayer for minute descriptions of these ; and proceed to the consideration of a disease, which is much more important, both from its frequency and severity, Impetigo. Great confusion, and no inconsiderable amount of practical error, has been introduced by Drs. Willan and Bateman into their descriptions of this and of another pustular disease, Porrigo,* by mistaking the varieties of the one for those of the other. Rayer, and following him Dr. Green, have contributed materially to improve our knowledge of these diseases, by pointing out the distinctive characters by which they may be discriminated from each other. Independently of other signs, " impetigo," says Dr. G. " is remarkably distinguished from the Tineas or Porrigos by the *non-contagiousness* of its nature, and by the form and character of its incrustations, which are moist, rugous, and continuous, not hard, dry, dimpled, and moulded, like those of true porrigo ;" and in another part, speaking of the two forms of Porrigo, he states, " in fact, no other disease of the skin is characterized by minute yellow pustules, whose contents concrete, almost as soon as they are visible, into little cup-shaped, hard, and extremely dry yellow scabs. When the incrustations are older they split into pieces, and often hang among the hair like the crusts of impetigo *granulata ;* but the absence of discharge, and the loss of the hair, always distinguish the porrigo from the impetiginous affection." Now the P. Larvalis (the crusta lactea of older authors) and the P. Favosa are certainly not contagious, and therefore they ought to be grouped among the species of Impetigo. Dr. Green correctly observes—

" Infants at the breast, and during the period of teething, are subject to a variety of impetigo *figurata,* which has long been familiarly known under the title of *crusta lactea,* and has been variously classed among the tineas and porrigos, by different writers, under the name of tinea *muciflua* of the face, porrigo *larvalis,* &c. The eruption in this affection is certainly impetiginous ; it consists, in fact, of a number of small superficial pale yellowish pustules, more or

---

* Rayer has abolished this term altogether, and has substituted that of Favus. Let it therefore be kept in mind, in perusing any extracts which we may make from Rayer's work, that his genus Favus corresponds with the genus Porrigo of Dr. Green.

less confluent, and collected into clusters. These bursting in four or five days, are succeeded by greenish yellow coloured incrustations, sometimes thin and laminated, at other times thick and rugous, exactly as in impetigo *figurata.* Fresh crops of pustules are evolved in the neighbourhood of the first clusters, which add to the extent of the disease, at the same time that a good deal of exudation is going on under the incrustations, and adding to their thickness. The surface of a portion of the scalp, of the ears and lips, and even the whole of the face, is often seen covered in this way with a kind of mask, whence the epithet *larvalis,* assumed by Willan as characterizing the disease." 137.

Again Rayer :—

"Impetigo *sparsa* may also attack the neck, ears, and hairy scalp. The tinea *granulata* of Alibert, and the porrigo *favosa* of Willan, signify one and the same variety of impetigo. Impetigo of the hairy scalp is occasionally met with among adults ; but the subjects it most usually attacks are children, and then it appears most frequently about the posterior parts of the head, the entire surface of which may be implicated. The disease appears in the shape of minute pustules of a yellowish-white colour, irregularly scattered over the hairy scalp, their centres traversed by a hair, and accompanied with a high degree of inflammation and excessive pruritus. In the course of from two to four days, these pustules pour forth a fluid that frequently agglutinates a number of hairs together, and dries into small brownish, or greyish rough and irregular crusts or masses, which M. Alibert has compared to fragments of roughly pounded mortar. These incrustations, become dry and friable, are detached from the skin, and remain adherent to the hair, which frequently appears filled with them. A very faint, sickly, and unpleasant smell is exhaled from the head, sometimes of so powerful a nature when all attention to cleanliness is neglected, that the whole atmosphere of a room is contaminated with it. Pediculi multiply rapidly, and swarm among the hair, which is rarely lost in this disease, but is very frequently matted or agglutinated into masses by the adhesive qualities of the discharge.

Impetigo of the hairy scalp scarcely lasts longer than some months, and it most frequently gets well with proper treatment in the course of a few weeks." 497.

As we wish to *debarrasser* this subject from the confusion in which it has been so long involved, we shall make another extract from Dr. Green, which, though long, will amply reward the reader's attention.

"By porrigo (the favus of Rayer) I understand a pustular and contagious disease of the skin, which commonly affects the scalp, but occasionally appears on other regions of the body also. This affection assumes two principal forms : in the one, the pustules characteristic of the disease are distinct and disseminated ; in the other they are confluent and collected into clusters, and ultimately into annular patches. In both varieties the pustules are *favi :* that is to say, small, rounded, pale yellow pustules, set, as it were, in a frame of epidermis, and containing a fluid, which almost immediately concretes into cup-shaped, straw-coloured scabs ; these go on increasing in thickness for many days, and at length, by cohering, compose a thick, hard, greyish yellow coloured incrustation, the surface of which presents a congeries of cup-shaped cavities like the sealed cells of a honey-comb ; this character however is frequently lost.

Under the head of porrigo, Willan, and those who have followed him, describe six different affections : porrigo *larvalis,* por. *favosa,* por. *furfurans,* por. *decalvans,* por. *lupinosa,* por. *scutulata.*

I have already had occasion to speak of two of these affections, porrigo *larvalis* and porrigo *favosa,* as species of another pustular eruption—impetigo, of which they are undoubtedly varieties. Porrigo *furfurans* may generally either be referred to one of the squamous affections—pityriasis, or be discovered to be an eczema or impetigo of the scalp. Porrigo *decalvans* is a title that does not seem chosen with Willan's usual felicity ; an effect of numerous diseases of the scalp being here assumed, as characteristic of a particular species. The state of baldness is a consequence of some affection of the bulbs which secrete the hair, and porrigo is only one of the forms of inflammation that attacks these organs, and either temporarily suspends or entirely destroys their functions. Finally, porrigo *lupinosa,* which I shall treat of under the title *favosa,* and porrigo *scutulata,* stand apart, and are distinguished from the whole of the other diseases which have been described under this name, in the circumstances of their common contagious nature, and the form of their pustules and primary incrustations." 152.

It is to be regretted that the English author, after this very able elucidation of a perplexed subject, should persist in retaining the epithet "Favosa" to one of the two forms of the genuine Porrigo. It would have been much wiser in him to have retained it by the epithet "Lupinosa," as he admits that his species, P. favosa, corresponds to Willan's "P. lupinosa." With this single exception, the remarks of Dr. Green are excellent. His observations on the treatment of Impetigo and Porrigo are certainly the most judicious which we have ever read. They are derived from sound pathological views.

"When impetigo appears as an acute inflammatory affection, which it frequently does, it must be treated upon the general principles which guide us in this class of complaints. In the milder forms, the antiphlogistic regimen and emollient tepid fomentations, as of milk and water, decoction of bran, &c. and light dressings of the unguent. oxid. zinci, or plumbi superacetatis ; in the severer, the local and even the general abstraction of blood, when there is nothing in the state of the constitution forbidding the practice, in addition to these measures, seldom fail to subdue the violence of the symptoms. The application of a couple of leeches, one behind each ear, in children of two or three years old, when the inflammation runs high, unremitting attention to cleanliness, the hair being clipped short when the scalp is the part affected, the constant use at first of emollient tepid fomentations, and subsequently of weak alkaline washes, whilst the parts affected are touched night and morning with the unguent. nitrat. hydrarg. properly diluted, combined with the exhibition, now and then, of some very gentle aperient medicine, will generally be found sufficient to bring to a happy conclusion either of the two varieties of impetigo (impetigo *larvalis,* imp. *granulata),* to which they are most subject." 141.

The vapour-douche is frequently of the greatest service in removing the incrustations, and in inducing a more healthy condition of the affected integuments. The administration of mild aperients, as the hydrargyrum cum cretâ, with rhubarb and carbonate of soda, will be found to facilitate the cure.

"When impetigo assumes a decidedly chronic type, the habitual use of a drink acidulated with the nitric or sulphuric acid, the occasional exhibition of an aperient dose of one of the neutral salts, as of the sulphate of magnesia, prolonged immersion in the tepid bath, and the topical application of a stimulating wash (the hydrocyanic acid properly diluted has been strongly recommended) may often be tried with decided advantage.

Should the inflamed surfaces continue indolent under this system of medicamentation, the more powerful influence of the hot air or vapour-bath, may be cautiously tried. These baths should be administered at a low temperature at first, and their effects carefully watched. If they do not cause too great a degree of re-action, and yet fail to bring about a decided improvement in the disease, the parts affected, after being cleared from scabs as much as possible by the use of the tepid douche and vapour-bath, may be touched with a weak solution of the nitrate of silver in distilled water. The citrine ointment is another stimulating application that is frequently employed under these circumstances with very good effect." 143.

In the very obstinate cases, Dr. Green has derived greater benefit from the use of the sulphur-fume bath, than from any other remedial means. [The cases related in the Periscope illustrate most satisfactorily the decided efficacy of this powerful agent.] The cautious administration of the arsenical solution is frequently attended with the most marked advantage, in some of the more intractable forms of Impetigo.* Rayer speaks highly of the good effects of the nitric acid, in doses of from half to a whole drachm in a pint of barley-water, taken daily in obstinate Impetigos. " It very seldom happens (says he) that this medicine is continued for a month or six weeks, without accomplishing a cure."

The treatment of genuine Porrigo is usually much more difficult than that of most of the forms of Impetigo. It appears to be more decidedly a local disease, and we quite agree with Dr. Green in the assertion, that—

" While various remedies are tried in succession, the most unremitting attention is to be paid to the ablution of the parts, and to the removal of incrustations and dead hair. This, indeed, is the sheet-anchor in the treatment of porrigo. In ninety-nine cases in a hundred, the disease will yield within three months to any plan of treatment adopted where this forms the principal feature, whilst every plan that can be pursued will fail nearly in the same proportion where it is neglected." 157.

Rayer, also, has strongly insisted on the necessity of unremitting attention to the daily ablution of all the affected integuments, and to the removal of all dry crusts and loosened hair by means of a small-tooth-comb. The head should never be shaved, as the irritation thus induced always aggravates the disease. The hair may be cut quite short enough with fine scissors.

M. Biett has recently recommended strongly the application of an ointment, composed of from a scruple to a drachm of the ioduret of sulphur, and an ounce of lard, in the chronic stage of the disease.

Mr. Wigan, in a late communication to the Medical Gazette, praises very highly the following mode of treatment:—The head being shaved, apply all over it the strong pyroligneous acid of Beaufoy (it is well known in the shops by the name of the " eleven acid"—one part to eleven of water, being of the strength of distilled vinegar) diluted with one-third its volume of water. The head should be kept wet with it for two or three minutes. Slight pain follows this application, and all the spots of the disease are rendered more visible, while the healthy portions are not so much affected. The diseased parts are now to be soaked with fresh applications of the acid, in its full strength, for a quarter of an hour. This medication is to be repeated at intervals of three or four days. Mr. W. assures the profession, that he has been extraordinarily successful in the treatment of Porrigo since he adopted the use of the acid, as now described, and that the cure is almost always effected in a short time. When Porrigo is very chronic, the following lotion has been used by some practitioners with decided advantage. An ounce of the compound spirit of ammonia, with two or three drachms of laudanum. It should be applied at night, and the citrine ointment applied during the day.

We pass over the subject of Ecthyma, as it cannot be easily mistaken for any other cutaneous disease, and as its history and treatment are sufficiently well detailed in Bateman's Synopsis.

*Papulæ.* The remarks of Rayer on the treatment of Lichen are so good, that we shall give them nearly entire.

---

* In a late Number of our valued cotemporary, the Edinburgh Medical and Surgical Journal, Dr. Thwaites has recorded several cases, in proof of the great efficacy of the internal use of Fowler's solution in Porrigo furfurans.

"Recourse must be had to the internal administration of the nitric, muriatic, and especially of the sulphuric acid in large doses. These mineral acids, when they seem to engender any considerable degree of irritability of the digestive organs, must however be replaced by some of the less active vegetable acids, such as the citric and acetic. As a final measure, and when the papulæ are extremely numerous, agglomerated and confluent in various places, as they are in the lichen *agrius*, if the patient be young and of good constitution, it may be necessary to detract blood once or oftener. General bloodletting is indeed an indispensable measure when the eruption is not confined to any determinate region of the body, such as the hand, face, &c. When blood is abstracted locally, the leeches employed must always be attached beyond the circle of the eruption; as without this precaution the irritation of their bites is apt to aggravate instead of relieving the local inflammation. All topical applications should be of the mildest description, and used cool or cold to the affected parts. Soothing washes, and gelatinous or mucilaginous baths, are generally useful when applied at low temperatures. Patients should, at the same time, be put upon the use of one of the acids which have been mentioned, combined as a sherbet, and should be advised to dress themselves with soft under garments, and such clothing as will not tend to excite or increase the heat of the body.

The effects of these various means ought to be seconded by a regimen of greater or less severity, according to the state of the constitution. Patients must at all events abstain from spiced food of every kind, spirituous liquors, in a word, from all that could possibly tend by its stimulating qualities upon the stomach, to produce at a later period any thing like a determination to the skin.

When lichen consists of several successive eruptions, and has assumed the chronic character, if the constitution of patients appears to have suffered from age, or any other cause, the practitioner should be careful to strengthen it as much as possible by the exhibition of tonics, and the recommendation of a suitable regimen, even before he thinks of attempting to treat the eruption.

When the disease is of very long standing, and affects the integuments deeply, the affected parts are often slightly anointed with the following ointment with the greatest advantage: ℞. Adipis suil. ℥j. Sulphur. sublim. ʒj. Potassæ subcarb. ʒss. Emollient temperate baths are to be used at the same time, and after some little interval has elapsed, they may even be taken slightly alkaline. Under similar circumstances I have frequently prescribed either of the following unguents with excellent effects: ℞, Adipis suil. ℥j. Calomelan. ʒj. Camphoræ, gr. xviij.—℞. Adipis suil. ℥j. Hydrarg. deuto-ioduret. gr. x. M. I have also occasionally succeeded in giving relief from the pruritus by cauterizing the affected parts of the skin very slightly with nitrate of silver, or by the use of vinegar and water as a lotion." 585.

And he adds:—

"When *chronic* lichen has withstood these various plans of treatment in an individual otherwise in good health, exempt from visceral affections, &c. when the disease is hereditary, is dispersed over a large surface of the body, and occasions distressing insomnia, we must then have recourse to some of the preparations of *arsenic*, and continue this class of medicines for a long time in graduated doses, it being always understood that they produce no manifestly injurious effects on the constitution. I have succeeded in removing several *circumscribed lichens* of old standing by the internal use of the solution of the arseniate of soda, and of sulphuretted alkaline inunctions. These remedies, however, it must be presumed, are only to be employed in the small number of cases in which all other means have been fruitlessly enforced, and the disease has attained such severity that patients, worn out and reduced to despair, insist on getting rid of their infirmity at all hazards. It is farther necessary, before prescribing these active medicines, to enquire particularly into the state of the digestive organs, only to increase the doses very gradually, and above all not to exceed from fifteen to twenty drops daily of Fowler's, or a drachm of Pearson's arsenical solution for an adult, in some mucilaginous fluid." 586.

The following practical remarks on the use of baths, by Dr. Green, are deserving of attention.

"The hot air or vapour bath, at a low temperature, may be cautiously adventured on; and if the eruption stands this kind of stimulus, we shall generally be able to cure the disease either by continuing the remedy, or recurring to the more powerful sulphur-fume bath. I have had repeated occasion to remark, that the hot air and sulphur-fume bath were always better borne, and produced more decidedly beneficial effects, when the parts affected were excoriated and moist from discharge; I have then no hesitation in prescribing this remedy, and always feel confident that it will prove beneficial. Even in the most chronic forms of this disease, a lax state of the bowels will be found of service." 181.

Baths are certainly more beneficial in cases of Prurigo than of Lichen. Dr. Green has given an excellent resumé of the means most worthy of trial in the former disease.

"The slighter cases of prurigo are successfully treated by a restricted diet, the occasional use of a gentle aperient, and prolonged immersion in the simple, weak alkaline, or sulphurous water bath, according to the degree and endurance of the eruption. In some cases it may be necessary to add the use of a diluent, acidulated with one of the mineral acids, or made slightly alkaline by the addition of half a drachm or a drachm of the subcarbonate of potash or soda, to each pint of the liquid, for a few days. A combination of sublimed sulphur and magnesia, or subcarbonate of soda, is also a good medicine in these cases; but the sulphur should be given in much smaller doses than those in which it is usually prescribed to produce its most beneficial effects; from two to four grains, with about ten or twelve of calcined magnesia, or subcarbonate of soda, twice a day, will be quite sufficient.

In none of its forms does this papular disease appear to be so much benefited by bloodletting as most of the other cutaneous inflammations. When blood has been drawn, however, even after the disease had merged into the chronic and and inveterate state, it has always shewn the buffy coat, or the large and firm crassamentum, characteristic of an inflammatory condition of the system. Though it may be mostly held unadvisable to bleed generally in this complaint, the application of a number of leeches around the seats of the local varieties will often be found a measure productive of great relief; and this practice seconded by due attention to regimen, will often bring the disease into a state to bear the stimulus of the vapour, hot-air, or sulphur-fume bath. When patients have been lowered by abstinence and the exhibition of a few doses of purgative medicine, I always recommend the vapour-bath to be tried at a low temperature at first, and if, to the temporary excitement which immediately follows the use of this remedy, an abatement of the symptoms succeeds, as usually happens, I then know that I can command the disease. I have even observed that the more inveterate the affection appeared to be, the more severely the parts affected were excoriated, the more they were complicated with discharging sero-purulent eruptions, the more certainly were they first amended and ultimately cured by perseverance in the use first of the vapour, and then of the hot air and sulphur-fume bath. Under the use of these means, the thickening of the skin and œdematous state of the subcutaneous cellular membrane are very speedily dissipated, and the withered, dry, and unperspiring surface rendered sleek and velvety to the touch, as it is in health." 188.

From the order of the Papulæ, we proceed to that of the Squamæ, or scaly diseases.

The classification of the squamæ adopted by Willan and Bateman is liable to several objections. This subject is admirably treated by Rayer.

"The number (says he) of squamous inflammations reckoned is six: Lepra, Psoriasis, Pityriasis, Pellagra, Acrodynia, and Scaly Syphilis. I shall treat of pellagra, and acrodynia when speaking of the cutaneous diseases peculiar to certain countries; and the history of squamous Syphilis will form part of that of the venereal disease in general. I have, farther, separated from the group of pityriasis, two varieties of cutaneous affection described by Willan under the heads pityriasis *versicolor*, and pityriasis *nigra*. These I denominate chloasma, and melasma, as I hold that they belong essentially to the class of pigmentary affections.

Mr. Plumbe and Dr. Duffin have proposed to unite the description of lepra to that of psoriasis. To place the distinguishing characters of these two diseases in greater relief, I have continued to describe them separately, although I still acknowledge the striking analogy that exists between their various symptoms. Inflammation of the reticular tissue and papillæ is the first and main feature of squamous affections. The squamæ are a secondary phenomenon, to which Willan attached too much importance when he assimilated icthyosis to this class of diseases."

"Two squamous inflammatory affections, lepra and psoriasis, make their appearance in the shape of small, hard, prominent and papular looking elevations, the tops of which soon become covered with squamæ of a dull white colour. These elevations unite, and then change into scaly patches of various forms and dimensions, which may either be in small numbers and limited to a single region of the body, or occur disseminated over the whole of its surface. In the latter case, the desquamation is frequently so copious that the bed and clothes of the patient become filled with dry and whitish-looking scales.

The dermis beneath the scaly patches is found red and inflamed; and inveterate squamous affections are always accompanied with chapping and a morbid increase of thickness in the skin.

Squamous inflammations occasionally remain confined to the points which they have first invaded, or they quit these to shew themselves on others. They sometimes cause itching, tingling, and an unpleasant sensation of heat, phenomena, all of which are constantly increased by such causes as tend to raise the external temperature of the body. These sensations are usually very violent in pityriasis. The insensible perspiration appears occasionally to be diminished in the places occupied by the squamæ.

The various forms of squamous inflammation frequently complicate each other —a new proof of their analogy. They are rarely combined with any other form of cutaneous affection."

"The squamous inflammations commonly require several months, and sometimes several years of treatment for their cure. They are the more obstinate, as they occupy a larger extent of surface, and as they are of longer standing.

With regard to treatment, few of the phlegmasiæ have so many points of resemblance as the squamous inflammations; to be satisfied of this it is enough to glance from the treatment of lepra to that of psoriasis." 616.

The genus icthyosis has been separated altogether from the order of the squamæ, because, in it, "the dermis is unaffected with any degree of redness, and is the seat of no morbid sensation." It has, therefore, been carried to a new order, established by Rayer, which he has denominated "hypertrophiæ," and which he thus characterizes :—

"The papillæ of the skin and the epidermis, the vascular rete, occasionally the entire substance of the skin, and even the subcutaneous tissues themselves, now and then occur of unusual thickness. This state may be either congenital or adventitious." 966.

The full propriety of this arrangement is questionable. We alluded to the subject in our former review of Rayer. Dr. A. T. Thomson makes the rather strange remark in the Cyclopædia, that " Ichthyosis has a much greater affinity to the papular than to the scaly eruptions."

There is perhaps no cutaneous disease, which merits the study of physicians more than that of Lepra, whether we reflect upon the mistakes which have been so frequently committed in its diagnosis, or the occasional extreme obstinacy of this affection against all remedial measures. The pathological characters of the disease also lead to some interesting inquiries. The circular shape of the squamous patches is well known to be the distinctive mark usually inculcated between Lepra and the cognate affection, Psoriasis; and some pathologists have therefore supposed that the superficial bloodvessels of the skin are disposed in small concentric circles; while others with more probability have attributed the circular form of the patches to the circumstance of their being developed at first as rounded elevations, the inflammation in spreading retaining its primary form. In the study of Lepra it is well to remember says Rayer, that it seems to be almost essentially a local disease, the morbid action not extending beyond the parts of the skin immediately involved.

It has been very generally stated, and perhaps with truth, that Lepra is now-a-days of more frequent occurrence, than it was fifty years ago. It is certainly not communicable by contact.

We shall probably allude at a future part of this article to the strange confusion which has been sometimes introduced into dermatology, by applying the appellation of Lepra, or Leprosy, to two essentially different diseases, viz. Greek Elephantiasis and Arabian Elephantiasis.

With respect to the prognosis of Lepra, we may observe, that, although not a dangerous disease, it is generally a very intractable one. When it attacks the aged, it is almost always incurable. Its hereditary forms, are, as might be anticipated, the most obstinate of any.

The treatment, therefore, of Lepra requires no inconsiderable practical skill. Remedial means are usually observed to be more efficacious in Spring and Summer, than in cold weather. Indeed many cases, especially in young people, appear to have a tendency to get well spontaneously during the continuance of warm weather.

Rayer evinces much judgment in his description of the treatment of Lepra. " When it is of a recent date, and extends over a large surface of the body, when the patches are affected with a painful pruritus, and the motions of the joints are impeded, the disease will be aggravated by sea bathing, by frictions or lotions with the preparations of sulphur, &c. which have been far too indiscriminately recommended in the treatment of diseases of the skin: blood-letting, on the contrary, and anointing the parts affected with cream, fresh butter, or sweet lard, give speedy relief. The vapour-bath, and the emollient or gelatinous bath may be employed as a principal or accessary means. The simple vapour-bath, occasionally proves sufficient of itself to cure recent lepra.

When the squamous patches are but very slightly inflamed, or of old standing, recourse is usually had to topical applications of a more or less exciting kind; before using these, however, it is always proper to cleanse the skin by means of fomentations, the tepid-bath, and gentle frictions, in case the scales adhere very firmly or lie in very thick layers. The use of stimulating washes, such as those composed of a mixture of spirits and water, or a solution of the sulphuret of potash, favours the removal of the scales, and often gives a favourable tendency to the progress of the patches. When the squamæ are detached, a little of the white pitch, tar, or weak nitrate of mercury ointment, may be rubbed over the affected parts, before the patient retires to rest for the night; next morning the skin must be washed with tepid water, or a weak saponaceous solution. By means of these topical applications, continued for several months, we occasionally succeed in restoring its natural texture to the skin, even after a course of internal medicines had been tried and failed. I have, however, obtained a still greater number of cures by the inunction of an ointment of the white precipitate, a dram of white precipitate to an ounce of hog's lard; a dram or even a dram and a half of this mixture may be rubbed in every day without any risk of exciting salivation; this is the external application I am in the habit of prescribing in the greater number of cases of lepra." 624.

The following remarks by Dr. Houghton in the Cyclopædia, are of great practical value.

" A great improvement in the treatment of skin-diseases generally has taken place since the utility of bloodletting has been recognized in reducing the inflammation from an active to a passive state. It will much facilitate the cure of even the smallest extent of the disease to begin by a good bleeding; but it is in cases where it has spread very generally over the body that the bleeding is to be mainly depended on, at least at the outset of the treatment.

Dr. Duffin, whose large experience of this remedy is very favourable to its use, after pointing out its striking usefulness in the circumstances adverted to above as peculiarly requiring it, adds, ' but supposing that there exist no general symptoms, still this mode of treatment is very often proper, were it had recourse to with no other view than to subdue the irritability of the skin or its extreme susceptibility to disease. But it has another good effect—it induces a state of the system that admits of being much sooner affected by the use of arsenic, when the active symptoms have been so far subdued as to allow of the employment of that medicine.' Its effect as a preparative is, indeed, the chief improvement we alluded to; and since it has been so employed, many remedies whose efficacy was much debated are now found decidedly useful, their exhibition being preceded by a bloodletting, and recourse being had to it during their use occasionally, if any symptom of the active inflammation re-appear. It would be obviously improper to employ it if the patient be in a debilitated state, the effects of a bad constitution, of the long duration of the disease, or of old age." 31.

In the more obstinate cases of the disease, the internal use of arsenic, or of the tincture of cantharides has been found strikingly efficacious, especially when the hot air, or sulphur fume baths have been employed at the same time. The liquor potassæ in gradually increased doses is also a medicine of approved credit.

Dr. Beck of Ipswich, recently published a clever Essay on Lepra, the object of which was to demonstrate the remedial powers of pitch, internally as well as externally administered, in this disease. Our readers will find an abstract of this Essay in the Number of this Review for last October.

The treatment of Psoriasis is essentially the same, as that of Lepra; indeed the two diseases appear to be, with the exception of the difference in the form of the squamous patches, essentially the same.

" By taking away," says Dr. Green, " a little blood from the arm, exhibiting a few aperient draughts, and soothing the irritable state of the surface by means of an emollient gruel bath in the first instance, and then resorting to the vapour and subsequently to the sulphur fume bath, I have never failed either in effecting the most signal amendment in the state of this rebellious disease, or in subduing it entirely.

The local varieties of psoriasis are to be treated in the same manner as the more general forms of the disease. A course of purgatives has often a marked and beneficial influence on several of them. The application of a number of leeches in the neighbourhood of the affected parts ought never to be neglected: they may be attached under the angle of the jaw or behind the ear in psoriasis *ophthalmica*, and around the wrist in psoriasis of the palm and back of the hand. The watery vapour and sulphur fume douche are often of essential service in these partial affections, as the steam and sulphur fume bath are in the more general disease.

When the parts are freed from squamæ by the use of the vapour bath or douche, a variety of unguents are often extremely useful in giving a favourable turn to the inflammation. I have already spoken of the liniment of the liquor potassæ. The unguentum hydrargyri nitratis of graduated strength, or a salve of the precipitated sub-muriate of the same metal, will often be found useful, especially in the *ophthalmic* and *labial* forms of the disease. In several of the other varieties, an ointment of the proto-ioduret, or deuto-ioduret of mercury, (12 grains to the ounce of lard,) or of the ioduret of sulphur, (15 to 20 grains to the ounce of lard,) will be found to infuse activity into the few indolent patches that sometimes remain about the elbows, having obstinately resisted the curative means that have been successfully used against the disease in other quarters." 225.

Arsenic is quite as efficacious in curing Psoriasis as Lepra, and the trial of it ought therefore never to be omitted in obstinate cases.

The division of the genus Pityriasis adopted by Willan has undergone considerable changes in Rayer's Treatise.

In the following remarks on its diagnosis from other cutaneous affections, it will be observed that two of Willan's species are transferred to another section of the classification.

"When pityriasis is compared with lepra the same points of difference are detected, with two distinguishing features in addition; the circular form of the patches of lepra, and their mode of recovery from the centre towards the circumference. The detachment of the cuticle in ichthyosis is not preceded by redness or morbid sensations of the skin. The desquamation that follows chronic lichen and eczema is preceded by the evolution of papulæ and vesicles. I shall by and by have occasion to contrast pityriasis with acrodynia and with pellagra, but I must here pause to expose the characters that distinguish it from chloasma (pityriasis *versicolor*, Willan), and from melasma (pityriasis nigra, Willan), diseases which I have felt called upon to transfer to another order, that, namely, of the adventitious *pigmentary discolorations*. In the first place, the most striking feature in the two diseases, last mentioned, is undoubtedly the change of colour presented by the skin; farther, if some degree of desquamation does take place at one period in the progress of these diseases, an habitual and abundant exfoliation of the cuticle forms no point in their history. Neither is there any of that serous exudation which I have mentioned when speaking of acute pityriasis; lastly, the ease with which chloasma is cured, and the deplorable resistance of pityriasis, in almost every instance, to remedial measures of every kind, show an essential difference in the nature of these two diseases. As to melasma (pityriasis *nigra*, Willan), when desquamation has once taken place, it seldom happens that this phenomenon and the other symptoms of the disease return with any intensity, or prove of any duration." 656.

Pityriasis is not usually a very troublesome complaint. We need not allude to the general principles which are to direct the physician in the treatment of it, as indeed they are applicable to every disease which affects the skin. The removal of all irritation, constitutional as well as local, and the correction of any derangement of the stomach, bowels and kidneys, are of invariable and most peremptory importance. In very obstinate cases, the internal use of the corrosive sublimate has succeeded, when all other means had failed; but this potent agent is much less uniformly successful than another, which fortunately is at the same time more simple and safe, we allude to the sulphur fume bath.

"I am happy," states Dr. Green, "to say, however, that I have never known one instance of pityriasis (and I have had cases under my care of thirty years' standing) which resisted a few exposures to the sulphur fume bath. I am in the habit, consequently, after prescribing a gentle aperient for a few days, to commence the use of this powerful remedial agent with as little delay as possible, and hitherto with complete success." 236.

Simple alkaline, or alkaline sulphureous lotions, the white precipitate ointment, and the vapour-douche, will almost always dissipate Pityriasis, when only a small surface is affected.

The two species of Pityriasis, designated P. versicolor and P. nigra in Willan's system, are carried by Rayer and Green to that part of their classifications which treats of the "alterations in the colour of the skin," as will be seen by referring to the tabular view given at the commencement of the present article. The P. versicolor, Chloasma, or, as it is vulgarly called, liver spots, is of very frequent occurrence in persons otherwise in perfect health. The colour of the spots varies, from that of the pale yellow of a withered leaf, to the deep yellow of rhubarb or saffron. They are usually scattered over the anterior parts of the chest and abdomen. Females are more subject to Chloasma than males; and the depth of the colour is generally observed to be increased during the catamenial periods.

"Sulphur, in one or another of its usual forms, appears to be a kind of specific in chloasma. Its spots disappear rapidly under the internal use of any of the common sulphureous mineral waters, as well as by the external employment of the same agents in the form of baths. When they resist these measures, the sulphur-fume bath will be found to dissipate them almost with a single application." 331.

And Rayer states—

"Chloasma is in general easily subdued by the use of sulphureous baths. Nevertheless I have seen several cases of a variety of this affection which is almost incurable, but which is happily of no real consequence. Almost the whole surface of the body was covered with large spots of a dirty-yellow colour, neither itchy nor furfuraceous, some of which it would have been impossible to cover with the palms of both hands joined together. In these rare cases, small streaks only, or isolated points of healthy skin, were seen between the spots. Several of the patients thus affected had been sent to various watering-places, but this without avail in so far as the affection of the skin was concerned, and almost always with temporary injury to their general health. These yellow discolorations of the skin resemble meladermia in their rebelliousness to treatment and their continuance." 949.

The Pityriasis nigra of Willan, or Melasma of Rayer, is much more uncommon than the species now described.

In our former review of Rayer's work, we promised to devote a page or two to the illustration of the two diseases, which have so unfortunately passed under the same name of Elephantiasis—the E. Græcorum, or Leprosy of the Middle Ages, and the E. Arabum. The former is truly a cutaneous disease, and ranks in the order of the Tubercula. It never ought to have been styled either Leprosy or Elephantiasis, as it neither has the characters of a scaly disease (Lepra), nor does it exhibit that hideous enlargement of any part of the body which can, with any propriety, be compared to the members of an elephant. It would have been well had Rayer insisted upon affixing a new name to it. He has thus described it.

"The Greek Elephantiasis is a serious chronic disease, characterized externally by shining and oily-looking dark patches, to which succeed irregular, slightly-prominent, softish, and at first, red and livid tubercles, which by and by assume a dusky or bronze colour; these usually continue long indolent; they may terminate in resolution or ulceration; their most common seat is the face. They also often appear on the palatine arch; but the nose and ears, swelled and hideously distorted, are the parts of all others which suffer most frequently."

"The cutaneous tubercles of elephantiasis are small, soft, round, reddish, or livid tumors, the size of which varies between that of a pea and that of an olive. They generally appear on every part of the face, on the nose and ears particularly, but are also, occasionally, though rarely, evolved on the legs only. When patients live under this infliction for a few years, the disease very commonly spreads to the whole body. The disease becomes even more and more marked. Of all the parts which are implicated, the face always bears the strongest traces of the havoc and deformity that characterise it. This seems to be generally puffed. The skin of the forehead, marked by numbers of deep transverse furrows, is beset with many tubercles, the superciliary ridges, swelled, and furrowed with oblique lines, are covered with nipple-like projections. The hair of the scalp, that of the eyebrows, and the cilia, are lost. The lips become thick and shining; the chin and concha of the ear enlarge, and become thickly covered with livid tumors ; the lobe and alæ of the nose, are in general even more seriously altered than the other parts of the face ; the nostrils are irregularly dilated ; lastly, the cheeks are swollen, and the whole of the features, enlarged and distorted by the puffing of the subcutaneous cellular membrane, acquire a character of the most frightful deformity." 735.

The tubercles of the Greek Elephantiasis are very seldom, or perhaps never seen on the trunk of the body. The upper and lower extremities are not unfrequently the seat of them ; but the face is the part most commonly affected. The tubercles, in course of time, either are resolved, or they suppurate and break. The sanious pus of the tubercles dries up speedily, and forms adhering brown or blackish scabs, which rarely rise above the level of the skin.

" The mucous membrane of the mouth, the velum palati, uvula, amygdalæ, pharynx, and nasal fossæ, very commonly also present tubercles, but less voluminous than those of the skin ; a longitudinal band of tubercles is frequently seen extending from the superior incisor teeth backwards along the roof of the mouth to the uvula. The lingual veins are occasionally observed to be varicose. An inflamed state of the pituitary membrane gives rise to the secretion of a sero-purulent fluid from the nostrils, to pain of the frontal sinuses, and finally to caries of the cartilages and turbinated bones of the nose. The voice becomes hoarse, nasal, and then is lost. The affections of the organ of hearing in elephantiasis do not extend beyond the external auricle. This part is enlarged, deformed, of a livid colour, and commonly beset with tubercles. The sense of smell, almost uniformly deranged even from the commencement of the disease, is always destroyed entirely when it has attained a certain stage ; that is to say, when the pituitary membrane, covered with tubercles, ulcerates, and pours out a profusion of fetid secretion. The eyes, except the deformity caused by the loss of the cilia, are seldom affected either externally or internally. Although the arch of the palate and the lining membrane of the mouth are frequently thickly beset with small tubercles, developed in the mucous follicles of this tissue, the sense of taste generally remains intact. The pharynx at length, is usually covered with tubercles, but the œsophagus is seldom thus affected. When the patient has not been put upon a lengthened course of purgatives or arsenical preparations, the stomach and intestines commonly perform their functions satisfactorily. Yet in the bodies of those who have died while labouring under Greek elephantiasis, the follicles of Peyer have been found very much developed, as well as intestinal tubercles ulcerated, or on the point of becoming so, small cicatrices, and enlarged or tubercular mesenteric glands (Larrey). The liver and spleen have not been observed to be morbidly affected. In accordance with the affection of the voice during life, a thickened state of the mucous folds of the larynx, tubercles upon the chordæ vocales, and occasionally ulcers which had destroyed the thyro-arytenoid ligaments, have been discovered after death. Neither is it uncommon to observe small ulcers of the mucous membrane of the trachea. The lungs have generally some crude or softened tubercles scattered through their substance. Three patients affected with elephantiasis, whose bodies I have examined, after their demise, presented this alteration of the lungs. Others, who have died at a less advanced stage of the disease, have shown unequivocal traces of pneumonia. The organs of circulation and of innervation present nothing peculiar, so long as the disease continues limited to the skin." 737.

The tubercular disease now described was first observed in Egypt, then in Italy during the time of Pompey, and subsequently it has been seen in all the quarters of the globe. During the middle ages, especially about the period of the Crusades, it spread over Europe like an epidemic; and lazarettos were established in almost every town for the reception of the "lepers." Since the beginning of the 17th century, it has disappeared from almost all the districts of our continent, and, at the present time, it seems to be limited to the inter-tropical regions. It has been studied by various authors within the last thirty years in Asia Minor, in Egypt and Abyssinia, in India, Sumatra, Ceylon, the West Coast of Africa, Madeira, Isle of France and Madagascar, West Indies, Brazils, and at New Orleans. Occasionally it has been witnessed in the South of France and in some parts of Spain. It is truly a cachexy, or disease connected with a depravation of the system, and appears to be, in some respects, a formidable aggravation of Ecthyma Cachecticum. Wherever it does occur, it attacks the poor and miserable infinitely more frequently than those who are in comfortable circumstances. An unhealthy climate, bad food and filthy habits, have certainly considerable influence in inducing this horrible disease. The Greek Elephantiasis has been supposed by some authors to be a mere modification of Syphilis. It is quite unnecessary to refute this opinion, but it deserves to be remembered in the study of the history of Syphilis, as given by the older authors in the 15th and 16th centuries, that the "Leprosy of the middle ages" was prevalent in these days. It is not improbable, therefore, that the characters of the new scourge, Syphilis, were not a little influenced by its attacking those who either were suffering, or had suffered from the Leprosy. We know nothing satisfactory as to the treatment of the Greek Elephantiasis. All the well-marked and severe cases of it are believed to be incurable. Those who are attacked with it before puberty commonly die from the 18th to the 25th year of their lives. The fatal termination is almost always owing to the sequelæ of inflammatory affections of the organs of respiration and of digestion. Removal from the climate, in which the disease has been acquired, is perhaps the most important part of the treatment; and when this step is taken, the adoption of all those means, dietetic as well as medicinal, which are most approved of for the amelioration of cachectic disease, ought to be vigorously adopted. Some East Indian practitioners have certainly derived much benefit in their treatment of the Greek Elephantiasis from the cautious administration of arsenic.

Allied in their nature and phenomena to, but certainly not identical with, the Greek Elephantiasis or Tubercular Leprosy of the middle ages, are the following endemic diseases which Rayer has grouped together under the term of elephantoid diseases: the Radesyge of the Norwegians;* the Lepra of Holstein, (Spedalsked); the Lepra Taurica, or Leprosy of the Cossacks; the Lepra Anæsthesiaca of the East Indies; the Ancient Leprosy of the Jews, and the Malum Mortuum of the middle ages. The admirably graphic description of the Lepra Anæsthesiaca by Dr. Robinson, in one of the volumes of the London Medico-chirurgical Transactions, will convey to the reader a tolerably correct notice of all these cognate diseases.

"One or two circumscribed spots or patches of a deeper colour, than that of the skin around them, appear on the feet or hands, and sometimes on the trunk and face; these spots are neither prominent nor depressed, they are shining and

---

* Some authors have considered this disease as a syphiloid affection. It presents a good many of the characters of the Greek Elephantiasis, and of confirmed secondary Syphilis together.

wrinkled; the wrinkles do not run into the surrounding healthy skin. The spots extend slowly until the skin of the legs and arms, and by degrees that of the whole body, when the disease is so extensive, is totally deprived of feeling. No perspiration takes place from the surfaces affected, neither are they itchy, nor painful, and it very seldom happens that they are swollen. In a more advanced stage of the disease the pulse becomes very slow, (fifty to sixty pulsations in a minute,) and soft without being small; constipation of the bowels follows; the toes and fingers are benumbed as though with cold, shining, slightly swelled, and somewhat stiff. The patient is indolent, slow in understanding the questions put to him, and seems to be constantly half asleep. The soles of the feet, and palms of the hands present hard and dry cracks; a furfuraceous matter is deposited under the nails, which raises them and occasions the skin around them to ulcerate. The legs and fore-arms swell; the skin is every where rough and chapped; at the same time ulcers form on the metacarpal and metatarsal articulations of the fingers, and toes, in the line of flexion, and in the corresponding parts of the articulations of the trunk, without any evident tumefaction or pain; pieces of skin half an inch in length become gangrenous and fall off, leaving the pale and flaccid muscles bare; these in their turn mortify, and by and by are also cast off. Different joints may be thus attacked and destroyed in succession, by the slow, but uninterrupted progress of this terrible disease, which renders those who are affected with it, objects of horror to all who approach them. The pains in this affection are not insupportable; the appetite is unaffected, and patients, horribly mutilated, sometimes live long without appearing to be disgusted with life. They are finally carried off by dysentery and diarrhœa. It deserves mention, that although *tubercular* elephantiasis sometimes shows itself during the course of elephantiasis *ancæthesiaca*, it is not necessarily consequent on it."

Very different from the Elephantiasis of the Greeks, and from all the other analogous affections, is that disease which has been designated Arabian Elephantiasis. The term Elephantiasis is sufficiently appropriate to this malady, as designating the remarkable and elephant-like enlargement of the affected parts; but the adjective appellative of the disease is a most absurd one, derived only from the circumstance of its having been first accurately described by Rhazes and Avicenna, Arabian authors.

This Elephantiasis is very properly detached by Rayer from the order, Tubercula, of cutaneous diseases. He has arranged it in a group which he calls Intumescentiæ, and which he thus characterises. "This group comprises several diseases with which the skin is not primarily affected, but which occasion hypertrophy of its different layers. These diseases, sometimes preceded or accompanied by fever at the commencement, are almost always followed by permanent *intumescence* or enlargement of the parts affected." 1112.

The parts usually affected are the limbs, especially the lower ones, the scrotum, and the labia pudendi; but the upper extremities, the face, the mammæ, the abdominal parietes, the verge of the anus, are occasionally subject to elephantoid enlargements.

Only one of these parts is commonly observed to be affected in the same patient. The disease begins in a manner not unlike to that in which diffuse inflammation of the cellular texture, or the Phlegmasia Dolens of puerperal women exhibits itself, and the morbid appearances discovered on dissection of patients who have died of Elephantiasis point out some degree of analogy between these diseases. In not a few cases which have of late years been minutely examined by some able pathologists in France, a morbid state of the veins of the lower limbs, the saphæna and the crural, has been detected. Rayer has reported a case, in which the anomalous development of one of the lower extremities coincided with a varicose state of the veins of the thigh, and he alludes to another case of Elephantiasis, in which he observed the contraction of one of the venæ saphenæ and the obliteration of the other. "In the left leg the vena saphena, laid bare along its whole extent, appeared in the form of a cylindrical cord of a yellowish-white colour, and not transparent, about a third less in size than the same vein in its natural state; the cavity of this vein was found almost obliterated at the point of junction between its middle and lower third; the vessel having been cut across in this place, a central point was distinguished upon each of the cut extremities into which a fine wire of the diameter of that which is usually inserted into silver catheters could be introduced, though not without difficulty; the calibre of this vessel had become in some sort, capillary, through an extent of about two inches; its sides being double their usual thickness; the vein cut across transversely in any point where it. was contracted, continued gaping in the same manner as an artery. The femoral vein towards its junction with the vena saphena, contained clots of recent formation; most of the other veins of this extremity presented no alteration. The vena saphena of the other leg, contained fibrinous clots of old formation, and adhering by their surface to the internal membrane of the vessel: the calibre of this vessel was not contracted, but its sides, like that of the right vena saphena, was thickened and resembled those of an artery." 1118.

M. Fabre found the vena saphena in a limb affected with Elephantiasis obstructed at several parts, and its walls greatly thickened. The posterior tibial vein also was obliterated in part of its course. The circumstance of this disease usually attacking those parts of the body in which the venous circulation is slowest and most liable to obstruction, viz. the lower limbs and scrotum, adds probability to the conjecture that inflammation of the veins may have some influence in producing it.

The lymphatic vessels and glands also of the affected parts are usually more or less diseased. The subcutaneous, cellular, and adipose tissues are always much hypertrophied and altered. "I have also found," says Rayer, "the cellular tissue infiltrated as it is in dropsies of long standing. M. Fabre has seen the subcutaneous cellular tissue converted into a thick, hard, almost fibro-cartilaginous layer presenting in several places small ossified plates, adhering so closely to the aponeurosis of the leg, and to the nerves and vessels which traverse it, that it was impossible to separate them. The sub-aponeurotic and intermuscular cellular tissue participated in the same alterations, but in a less degree. In a woman who died in the Hôpital de la Charité, in 1820, whose lower limbs were affected with elephantiasis, M. Andral found, under the skin, and in the place of the muscles of this limb which were reduced to some thin discoloured shreds, an enormous mass of hard, condensed cellular tissue, with cavities here and there filled with serum, and partaking, in more than one place, of all the qualities of cartilage (Precis d'anatomie pathologique, t. i. p. 277.)" 1117.

The etiology of Elephantiasis is very imperfectly understood. As a general remark, it may be observed, that certain alterations of the veins, (varyx, phlebitis, contraction, obliteration, &c.) and various forms of cutaneous inflammation, (erysipelas, eczema, lichen, ulcers,) are the diseases which have been the most frequently observed before the developement of Arabian Elephantiasis or during its course.

From the preceding observations on this disease, it must be obvious that it is in almost every respect essentially different from the disease to which the appellation of Greek Elephantiasis has been so absurdly given. The latter is an infinitely more formidable malady than the former; the one is truly a constitutional cachexy, the other appears to be much more local in its origin and seat. With regard to the treatment of the Arabian Elephantiasis, rational hopes of a cure may frequently be entertained. General and topical bleedings, emollient fomentations, and entire rest, are the most judicious remedial measures at the commencement of the disease; and afterwards friction, and regulated compression, by means of straps of plaster and of rollers, will be found of the greatest service. Internal medicines have not been found of much use in Arabian Elephantiasis.

